# Svep1 stabilises developmental vascular anastomosis in reduced flow conditions

**DOI:** 10.1242/dev.199858

**Published:** 2022-03-25

**Authors:** Baptiste Coxam, Russell T. Collins, Melina Hußmann, Yvonne Huisman, Katja Meier, Simone Jung, Eireen Bartels-Klein, Anna Szymborska, Lise Finotto, Christian S. M. Helker, Didier Y. R. Stainier, Stefan Schulte-Merker, Holger Gerhardt

**Affiliations:** 1 Integrative Vascular Biology Lab, Max-Delbrück Center for Molecular Medicine in the Helmholtz Association (MDC), Robert-Rössle-Strasse 10, Berlin 13125, Germany; 2 DZHK (German Center for Cardiovascular Research), Berlin 10785, Germany; 3 Institute of Cardiovascular Organogenesis and Regeneration, Faculty of Medicine, WWU Münster, Mendelstraße 7, 48149 Münster, Germany; 4 Vascular Patterning Laboratory, Center for Cancer Biology, VIB, Leuven 3000, Belgium; 5 Vascular Patterning Laboratory, Department of Oncology, KU Leuven, Leuven 3000, Belgium; 6 Department of Developmental Genetics, Max Planck Institute for Heart and Lung Research, Bad Nauheim 61231, Germany; 7 Berlin Institute of Health (BIH), Berlin 10178, Germany

**Keywords:** Anastomosis, Angiogenesis, Vegfa/Vegfr signalling, Zebrafish

## Abstract

Molecular mechanisms controlling the formation, stabilisation and maintenance of blood vessel connections remain poorly defined. Here, we identify blood flow and the large extracellular protein Svep1 as co-modulators of vessel anastomosis during developmental angiogenesis in zebrafish embryos. Both loss of Svep1 and blood flow reduction contribute to defective anastomosis of intersegmental vessels. The reduced formation and lumenisation of the dorsal longitudinal anastomotic vessel (DLAV) is associated with a compensatory increase in Vegfa/Vegfr pERK signalling, concomittant expansion of *apelin*-positive tip cells, but reduced expression of *klf2a*. Experimentally, further increasing Vegfa/Vegfr signalling can rescue the DLAV formation and lumenisation defects, whereas its inhibition dramatically exacerbates the loss of connectivity. Mechanistically, our results suggest that flow and Svep1 co-regulate the stabilisation of vascular connections, in part by modulating the Vegfa/Vegfr signalling pathway.

## Abstract (precis)

**Summary:** Blood flow and the large extracellular matrix protein Svep1 jointly regulate vessel anastomosis during developmental angiogenesis in zebrafish embryos partly by modulating the Vegfa/Vegfr signalling pathway.

## INTRODUCTION

Angiogenesis defines the formation of new vessels from pre-existing ones and is a stepwise process that leads to the establishment of a perfused network of arteries and veins that are optimally organised to serve the metabolic needs of the developing embryo. In zebrafish embryos, the initial coalescence of endothelial progenitor cells in a process termed vasculogenesis shapes the two main axial blood vessels, namely the dorsal aorta (DA) and the posterior cardinal vein (PCV). Later, angiogenic sprouts emerge from the DA. These multicellular sprouts are composed of a leading tip cell followed by stalk cells (Hogan and Schulte-Merker, 2017), and migrate dorsally in between vertical somite boundaries to form the intersegmental vessels (ISVs) (Isogai et al., 2003). The proliferation of endothelial cells in ISVs and their migration to the dorsal part of the embryo is driven by Vegfa signalling, through binding to the zebrafish VEGFR2 orthologue Kdr and its ohnolog Kdrl (Covassin et al., 2006, 2009; Bussmann et al., 2010, 2008; Shin et al., 2016; Bahary et al., 2007). Activation of Kdrl signalling in the leading ISV endothelial cell (tip), mediated in large part via the phosphorylation of the serine/threonine kinase ERK1/2, promotes migratory behaviour. In parallel, activation of Kdrl signalling in the tip cell leads to Notch-mediated inhibition of Kdrl signalling in trailing cells (stalks), preventing their conversion into tip cells and promoting their proliferation to support ISV expansion (Shin et al., 2016; Geudens and Gerhardt, 2011; Siekmann and Lawson, 2007). At ∼30-32 h post-fertilisation (hpf), the leading tip cells of the ISVs start to anastomose with their ipsilateral neighbours, a process that ultimately leads to the formation of the dorsal longitudinal anastomotic vessel (DLAV), dorsal to the neural tube (Betz et al., 2016; Zygmunt et al., 2012). The DLAV is initially a paired bilateral structure that is fully lumenised by 48 hpf, but subsequently both sides progressively connect to form a complex plexus (Isogai et al., 2003; Zygmunt et al., 2012). Zygmunt and colleagues (Zygmunt et al., 2012) demonstrated that maturation of the DLAV plexus is regulated by flow and Vegfr signalling after 48 hpf. However, although they show that flow is dispensable for the initial formation of the DLAV, little is known about the cellular mechanism driving the anastomosis of ipsilateral ISVs and the lumenisation of the DLAV segments during DLAV formation (32-48 hpf).

Recent reports have characterised the importance of Svep1 (also known as Polydom), a secreted extracellular matrix (ECM) protein that can mediate cell-to-substrate adhesion *in vitro* in an integrin α9β1-dependent manner (Sato-Nishiuchi et al., 2012), as a regulator of secondary angiogenesis (Karpanen et al., 2017; Morooka et al., 2017). In zebrafish, loss-of-function *svep1* mutants exhibit a reduced number of venous and lymphatic precursors (parachordal lymphangioblasts; PLs) emerging from the PCV during secondary angiogenesis (from 32 hpf). In addition, PLs show reduced migration from the horizontal myoseptum. Both these defects lead to an increased number of arterial ISVs (aISVs) and a severe reduction in the lymphatic trunk vasculature. Here, we uncover a previously unreported and distinct role for Svep1 in the regulation of DLAV formation under reduced flow conditions, acting in part through the modulation of Vegfa/Vegfr signalling in endothelial cells.

## RESULTS

### *svep1* mutant and morphant zebrafish embryos exhibit vascular anastomosis defects

**Fig. 1.**
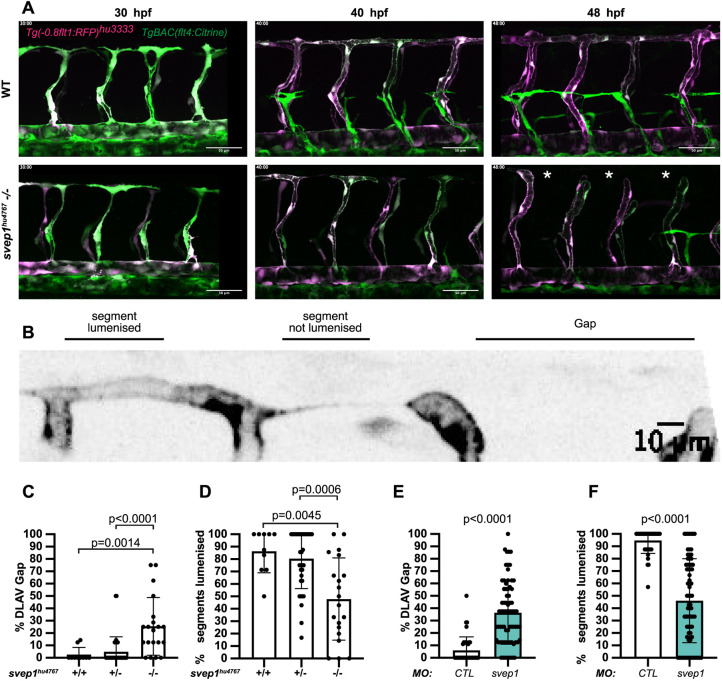
***svep1* mutant and morphant zebrafish embryos exhibit vascular anastomosis defects.** (A) Stills from time-lapse movie of *Tg(-0.8flt1:RFP)^hu3333^; TgBAC(flt4:Citrine)* embryos injected with *MO-CTL* (5 ng) or *MO-svep1* (5 ng) and treated with 1× (0.014%) tricaine from 30 to 48 hpf. White asterisks indicate gaps in the DLAV. (B) Still from a time-lapse movie of a *Tg(-0.8flt1:RFP)^hu3333^; TgBAC(flt4:Citrine)* embryo injected with *MO-CTL* (5 ng) exhibiting a gap in the DLAV between two adjacent ISVs. Side view, dorsal side left. (C) Bilateral quantifications of the percentage of gaps in the DLAV at 48 hpf in WT (*n*=5), *svep1^hu4767^* heterozygous (*n*=18) and *svep1^hu4767^* homozygous embryos (*n*=10) treated with 1× tricaine (0.014%) from 30 to 48 hpf (*N*=3). (D) Bilateral quantifications of the percentage of lumenised segments in the DLAV at 48 hpf in WT (*n*=5), *svep1^hu4767^* heterozygous (*n*=18) and *svep1^hu4767^* homozygous embryos (*n*=10) treated with 1× tricaine (0.014%) from 30 to 48 hpf (*N*=3). (E) Bilateral quantifications of the percentage of gaps in the DLAV at 48 hpf in embryos injected with *MO-CTL* (5 ng) (*n*=20) and *MO-svep1* (5 ng) (*n*=36) and treated with 1× tricaine (0.014%) from 30 to 48 hpf (*N*=6). (F) Bilateral quantifications of the percentage of lumenised segments in the DLAV at 48 hpf in embryos injected with *MO-CTL* (5 ng) (*n*=20) and *MO-svep1* (5 ng) (*n*=36) and treated with 1× tricaine (0.014%) from 30 to 48 hpf (*N*=6). Data are mean±s.d. Mann–Whitney test. Scale bars: 50 μm (A); 10 μm (B).

While imaging angiogenesis in the trunk of *svep1^hu4767^* loss-of-function mutants (Karpanen et al., 2017) from 30 to 48 hpf, we noticed that a significant number of primary angiogenic sprouts failed to anastomose with their ipsilateral neighbours (Fig. 1A) (Movies 1,2). In the majority of cases (74%±33; mean±s.d.), the DLAV gaps arose following the regression of a pre-existing connection between ipsilateral neighbouring ISVs, rather than an absence of connection (*N*=6 experiments, *n*=18 mutants). In addition, only a minority of these connections (13%, *N*=6 experiments, *n*=17 mutants) were transiently lumenised before regressing. These results suggest that, in this context, *svep1* loss-of-function negatively affects the stabilisation of vascular connections between neighbouring sprouts.

We quantified the number of gaps in the DLAV and the lumenisation status of existing DLAV segments at 48 hpf, a time at which the DLAV is considered fully formed and almost fully lumenised in wild-type (WT) zebrafish embryos (Isogai et al., 2003; Zygmunt et al., 2012). *svep1^hu4767^* mutants exhibited a significantly increased number of gaps in their DLAV at 48 hpf (25.5%±23.4 versus 2.6%±5.6 in their WT siblings) and a significant decrease of lumenised DLAV segments (47.8%±33.1 versus 86.4%±17.3) (Fig. 1B-D). These phenotypes were also observed in *svep1* morphants compared with control morphants (DLAV gaps: 36.3%±25.4 versus 6%±10.9; lumenised DLAV segments: 46.1%±33.8 versus 94.7±10.5) (Fig. 1E,F). In addition, although the expressivity of the morphant phenotype in different zebrafish transgenic lines varied markedly, we observed a statistically significant difference between *svep1* and control morphants in all cases (Fig. S1A,B). Finally, we found a variable expressivity of the *svep1^hu4767^* homozygous mutant DLAV phenotype between clutches (Fig. S1C-F).

### *svep1* loss-of-function sensitises angiogenic remodelling to reduced blood flow

**Fig. 2.**
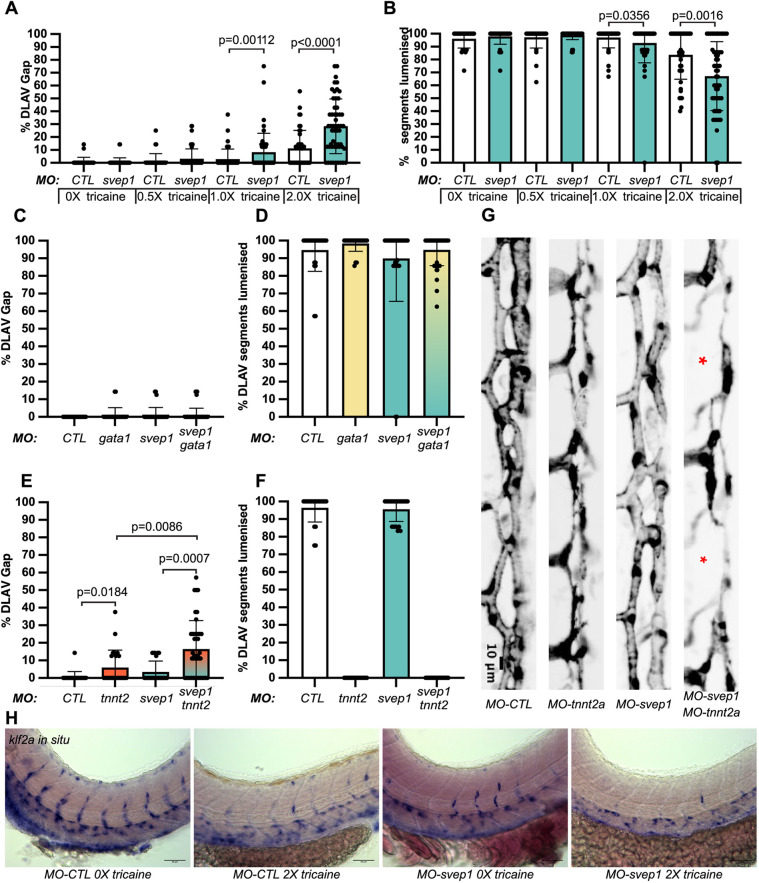
***svep1* loss-of-function sensitises angiogenic remodelling to reduced blood flow.** (A) Bilateral quantifications of the percentage of gaps in the DLAV at 48 hpf in embryos injected with *MO-CTL* (5 ng) and *MO-svep1* (5 ng) and treated with 0× (*n*=14 *MO-CTL*, *n*=20 *MO-svep1*), 0.5× (*n*=16 *MO-CTL*, *n*=24 *MO-svep1*), 1× (*n*=22 *MO-CTL*, *n*=27 *MO-svep1*) or 2× (*n*=21 *MO-CTL*, *n*=27 *MO-svep1*) from 30 to 48 hpf (*N*=3). (B) Bilateral quantifications of the percentage of lumenised segments in the DLAV at 48 hpf in embryos injected with *MO-CTL* (5 ng) and *MO-svep1* (5 ng) and treated with 0× (*n*=14 *MO-CTL*, *n*=20 *MO-svep1*), 0.5× (*n*=16 *MO-CTL*, *n*=24 *MO-svep1*), 1× (*n*=22 *MO-CTL*, *n*=27 *MO-svep1*) or 2× (*n*=21 *MO-CTL*, *n*=27 *MO-svep1*) from 30 to 48 hpf (*N*=3). (C) Bilateral quantifications of the percentage of gaps in the DLAV at 48 hpf in embryos injected with *MO-CTL* (5 ng) (*n*=13), *MO-gata1* (8 ng) (*n*=12), *MO-svep1* (5 ng) (*n*=16) and *MO-gata1* (8 ng)/*MO-svep1* (5 ng) (*n*=25) (*N*=3). (D) Bilateral quantifications of the percentage of lumenised segments in the DLAV at 48 hpf in embryos injected with *MO-CTL* (5 ng) (*n*=13), *MO-gata1* (8 ng) (*n*=12), *MO-svep1* (5 ng) (*n*=16) and *MO-gata1* (8 ng)/*MO-svep1* (5 ng) (*n*=25) (*N*=3). (E) Bilateral quantifications of the percentage of gaps in the DLAV at 48 hpf in embryos injected with *MO-CTL* (5 ng) (*n*=11), *MO-tnnt2a* (4 ng) (*n*=12), *MO-svep1* (5 ng) (*n*=12) and *MO-tnnt2a* (4 ng)/MO-svep1 (5 ng) (*n*=21) (*N*=3). (F) Bilateral quantifications of the percentage of lumenised segments in the DLAV at 48 hpf in embryos injected with *MO-CTL* (5 ng) (*n*=11), *MO-tnnt2a* (8 ng) (*n*=12), *MO-svep1* (5 ng) (*n*=12) and *MO-tnnt2a* (4 ng)/*MO-svep1* (5 ng) (*n*=21) (*N*=3). (G) Maximum intensity projection of dorsal view of the DLAV from representative embryos injected with *MO-CTL* (5 ng) (*n*=11), *MO-tnnt2a* (8 ng) (*n*=12), *MO-svep1* (5 ng) (*n*=12) and *MO-tnnt2a* (4 ng)/*MO-svep1* (5 ng) (*n*=21) embryos at 48 hpf. Red asterisks indicate gaps. (H) Bright-field images of *klf2a in situ* hybridisation of 48 hpf embryos injected with *MO-CTL* or *MO-svep1* and treated with 0× or 2× tricaine from 30 to 48 hpf. Data are mean±s.d. Mann–Whitney test. Scale bars: 10 μm (G); 50 μm (H).

Surprisingly, we could not detect any anastomosis defects at the DLAV in *svep1^hu4767^* mutants and morphants when imaged at 48 hpf, although they continued to exhibit the previously reported PL phenotypes (Karpanen et al., 2017) (PLs at the horizontal myoseptum: 47.6%±17.5 versus 80.2%±19.4% in WT clutch mates; aISVs: 65.4%±15.1 versus 49.3%±15 in WT siblings; *N*=4, *n*=12 mutants, *n*=25 WT). The DLAV phenotypes instead only occurred in mutant embryos that were imaged live from 30 to 48 hpf. Upon closer inspection, we found that treatment with tricaine (tricaine mesylate – MS222), a muscle relaxant commonly used to immobilise zebrafish embryos, led to a dose-dependent emergence of the DLAV phenotypes in *svep1* loss-of-function morphants (Fig. 2A,B) following treatment with a concentration of 1× (0.014%) or above from 30 to 48 hpf.

In addition, removal of tricaine from mutant embryos at 48 hpf led to a significant recovery of the DLAV vasculature at 72 hpf, with only 27%±26 of the DLAV gaps still present at that stage (Fig. S2A,B).

Previous work has revealed that *svep1* loss-of-function is associated with cardiac defects (Karpanen et al., 2017). To test whether *svep1* loss-of-function further sensitises embryos to blood flow reduction, we quantified heartbeats per minute in control and *svep1* morphants and found no differences in embryos treated with 1× (0.014%) or 2× (0.028%) tricaine from 30 to 48 hpf (Fig. S2C). However, we found that mean blood flow speed was significantly decreased in *svep1* morphants compared with control morphants when treated with 1× tricaine (MO-CTL 5 ng: 679 μm/s ±205; MO-svep1 5 ng: 521 μm/s±190). This suggest that the cardiac phenotype detected in *svep1* loss-of-function embryos exacerbates the blood flow reduction induced by tricaine treatment.

To further characterise the phenotype we decided to begin our investigation at 30 hpf, as this time point marks the beginning of ISV ipsilateral anastomosis in most embryos (Fig. 1A). As tricaine treatment leads to a reduction of blood flow speed (Geudens et al., 2019), we investigated whether a general blood flow speed reduction or a reduction of erythrocyte-dependent shear stress was responsible for the DLAV phenotype in *svep1* morphants. In the absence of tricaine treatment, joint inhibition of *svep1* function and erythrocyte formation, using *svep1* and *gata1* morpholinos, did not lead to any DLAV phenotypes (Fig. 2C,D). However, in the absence of tricaine treatment, abolition of cardiac function, and thus blood flow, using a morpholino against *troponin T type 2a* (*tnnt2a*) led to a DLAV phenotype in *svep1* morphants (Fig. 2E-G) (16.4%±16.2 versus 3.5±6.2 in *svep1* morphants only). Abolition of blood flow in *svep1* homozygous mutants led to an increase in the proportion of fish exhibiting a strong DLAV phenotype (30% compared with 5.3% of WT clutch mate) (Fig. S2F). These results suggest that *svep1* loss-of-function has an additive effect in affecting blood vessel anastomosis when blood flow is reduced, and that *svep1* loss-of-function further sensitises primary ISVs anastomosis to reduced blood flow or pressure, but not to reduced shear stress.

*klf2a*, a key transcription factor gene upregulated by laminar shear stress in endothelial cells (Parmar et al., 2006), also showed reduced expression after tricaine treatment or *svep1* knockdown, and almost complete absence from ISVs when *svep1* knockdown was combined with tricaine (Fig. 2H). In contrast to ISVs and DLAV, the major axial vessels showed no reduction in *klf2a* expression, further suggesting that shear sensing in general is not affected by tricaine and/or *svep1* loss-of-function.

In addition, we observed that *svep1* loss-of-function significantly increases the percentage of short ISVs in *tnnt2a* morphants at 48 hpf (14.6%±10.5 versus 4.8%±7.6 in *tnnt2a* morphants only). This led us to investigate the importance of *svep1* in the regulation of angiogenic sprout identity and behaviour under reduced flow conditions.

### Svep1 is expressed in neurons of the neural tube and its expression is flow dependent

**Fig. 3.**
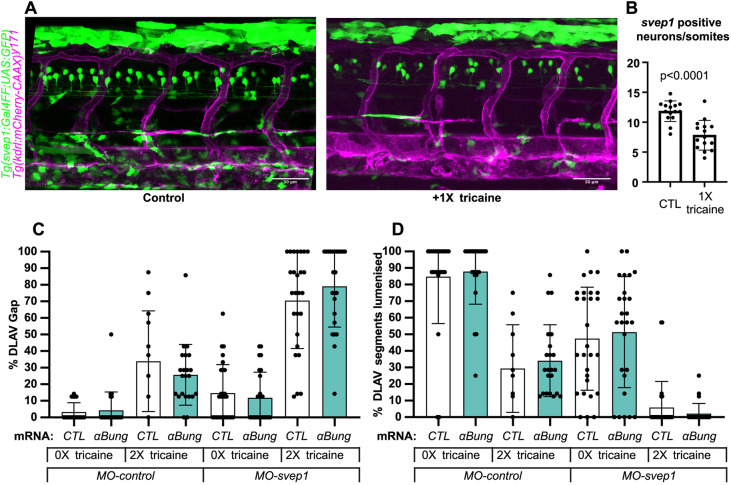
***svep1* is expressed in neurons in the neural tube.** (A) Representative images of 48 hpf *Tg(svep1:Gal4FF; UAS:eGFP); Tg(kdrl:mcherry-CAAX)^y171^* embryos with or without treatment with 1× (0.0168%) tricaine from 30 to 48 hpf. (B) Quantification of average numbers of *Tg(svep1:Gal4FF; UAS:eGFP)*-positive neurons in the neural tube area of 48 hpf embryos with or without treatment with 1× (0.0168%) tricaine from 30 to 48 hpf (*N*=3, *n*=15 controls, *n*=16 treated). (C) Bilateral quantifications of the percentage of gaps in the DLAV at 48 hpf in embryos injected with control mRNA (GFP, 50 pg) or alpha-bungarotoxin (*αBung*) mRNA, *MO-CTL* (5 ng) or *MO-svep1* (5 ng), and treated with 0× or 2× tricaine from 30-48 hpf. (*N*=2, *n*=24-28). (D) Bilateral quantifications of the percentage of lumenised segments in the DLAV at 48 hpf in embryos injected with control mRNA (GFP, 50 pg) or *αBung* mRNA, *MO-CTL* (5 ng) or *MO-svep1* (5 ng) and treated with 0× or 2× tricaine from 30 to 48 hpf (*N*=2, *n*=24-28). Data are mean±s.d. Mann–Whitney test. Scale bars: 50 μm (A).

Imaging of the *svep1* reporter line *Tg(svep1:Gal4FF;UAS:GFP)* (Karpanen et al., 2017) at 48 hpf showed strong GFP expression in dorsal epithelial cells above the neural tube, and in individual neurons of the neural tube (Fig. 3A; Fig. S3A,B). Treatment with 0.0168% tricaine from 30 to 48 hpf led to a significant reduction in *svep1* expression throughout the trunk area, particularly in the neural tube at 48 hpf (Fig. 3B). In addition, tricaine treatment between 30 and 48 hpf led to a significant reduction in endogenous *svep1* mRNA expression within the neural tube and ventral somite boundary (Fig. S3C), suggesting that blood flow not only sensitises angiogenic sprouts to *svep1* downregulation, but also directly affects *svep1* expression.

Given that tricaine, in addition to lowering cardiac function, has additional effects on neural voltage-gated sodium channels (Attili and Hughes, 2014), and that we observed reduced neural expression of *svep1*, an alternative explanation for the DLAV phenotype could be tricaine interfering with neuronal function, which in turn augments the effects of *svep1* knockdown. To test this possibility, we selectively blocked neuronal activity but not cardiac function by alpha-bungarotoxin mRNA injection (Swinburne et al., 2015), and quantified DLAV gap formation and lumenisation in comparison with, and in combination with, tricaine treatment (Fig. 3C,D). Alpha-bungarotoxin had no effect on DLAV formation or lumenisation on its own, and did not augment the tricaine and *svep1* loss-of-function effects. These results suggest that the observed sensitisation to *svep1* loss-of-function by tricaine is attributable to its effects on blood flow.

### *svep1* loss-of-function leads to a defect in tip/stalk cell specification in primary angiogenic sprouts

**Fig. 4.**
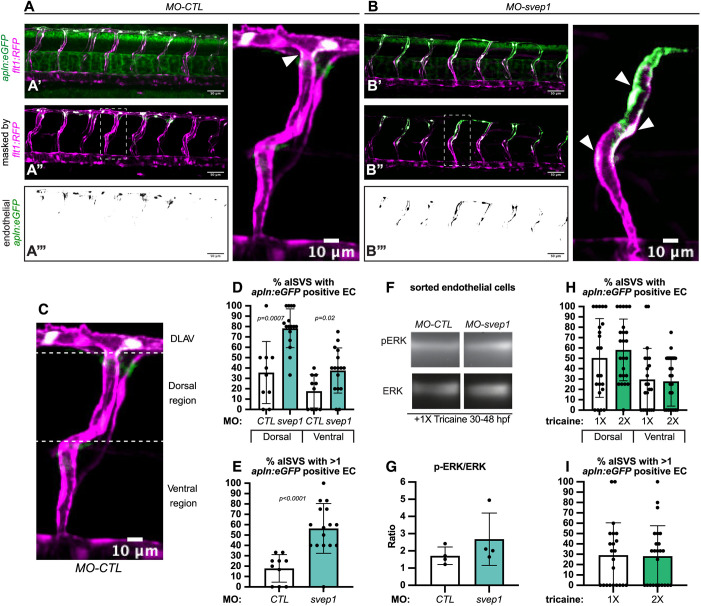
***svep1* loss-of-function leads to a defect in tip/stalk cell specification in primary angiogenic sprouts.** (A) Maximum intensity projection of a representative *TgBAC(apln:eGFP)^bns157^, Tg(-0.8flt1:RFP)^hu5333^ MO-CTL* (5 ng) embryo. A′ shows the unprocessed maximum intensity projection, A″ shows the GFP signal volume-masked by the RFP signal, to limit detection to the endothelium and A‴ shows the resulting endothelial GFP signal only. Panel on right is magnification of boxed area in A″. (B) Maximum intensity projection of a representative *TgBAC(apln:eGFP)^bns157^, Tg(-0.8flt1:RFP)^hu5333^MO-CTL* (5 ng) embryo. B′ shows the unprocessed maximum intensity projection, B″ shows the GFP signal volume-masked by the RFP signal, to limit detection to the endothelium and B‴ shows the resulting endothelial GFP signal only. Panel on right is magnification of boxed area in B″. White arrowheads indicate *apln:eGFP* expression in ISVs in A and B. (C) Maximum intensity projection of a MO-CTL (5 ng) aISV at 48 hpf, highlighting the ventral and dorsal region used for further quantifications in D and E. (D) Quantification of the percentage of aISVs with *apln:eGFP*-positive endothelial cells in the dorsal and ventral regions in 48 hpf MO-CTL (5 ng) (*n*=10) and *MO-svep1*(5 ng) (*n*=16) morphant embryos treated with 1× (0.014%) tricaine from 30 to 48 hpf (*N*=3). (E) Quantification of the percentage of aISVs with more than one *apln:eGFP*-positive endothelial cell in 48 hpf *MO-CTL* (5 ng) (*n*=10) and *MO-svep1* (5 ng) (*n*=16) morphant embryos treated with 1× (0.014%) tricaine from 30 to 48 hpf (*N*=3). (F) Representative image of p-ERK and ERK levels in FACS sorted endothelial cells from *MO-CTL* (5 ng) and *MO-svep1* (5 ng) morphants at 48 hpf treated with 1× (0.014%) tricaine from 30 to 48 hpf (*N*=4). (G) Quantification of p-ERK in FACS sorted endothelial cells of *MO-CTL* (5 ng) and *MO-svep1* (5 ng) morphants at 48 hpf, treated with 1× (0.014%) tricaine from 30 to 48 hpf. Expression levels were normalised to total ERK levels (*N*=4). (H) Quantification of the percentage of aISVs with *apln:eGFP*-positive endothelial cells in the dorsal and ventral regions in 48 hpf embryos treated with 1× (0.014%) (*n*=22) or 2× (0.028%) (*n*=24) tricaine from 30 to 48 hpf (*N*=3). (I) Quantification of the percentage of aISVs with more than one *apln:eGFP*-positive endothelial cell in 48 hpf embryos treated with 1× (0.014%) (*n*=22) or 2× (0.028%) (*n*=24) tricaine from 30 to 48 hpf (*N*=3). Data are mean±s.d. Mann–Whitney test. Scale bars: 50 μm (A′-A‴,B′-B‴); 10 μm (A,B, magnification, C).

The formation of the DLAV is initiated by the anastomosis of ipsilateral arterial sprouts, led by a tip cell (Hogan and Schulte-Merker, 2017). To investigate tip cell identity in the zebrafish trunk, we took advantage of the recently published *Tg(apln:eGFP)* reporter line (Marín-Juez et al., 2019), in which endothelial tip cells are highlighted by eGFP expression at 48 hpf. Following treatment with tricaine from 30 to 48 hpf, *svep1* morphants exhibited an expansion of *apelin* (*apln*)*+* endothelial cells in ISVs, whereas control morphants predominantly presented *apln+* cells in the dorsal-most region of the trunk vasculature, consistent with a contribution of tip cells to the formation of the DLAV (Fig. 4A-D). In addition, *svep1* morphants presented an overall increase in *apln+* cells per ISV (56.5%±24 versus 17.8%±13.3 in control morphants) (Fig. 4E).

As tip cell identity is in part characterised by increased levels of p-ERK downstream of Vegfa/Vegfr signalling, we next investigated this pathway activation in *svep1* morphants. Interestingly, sorted total endothelial cells from 48 hpf *svep1* morphants, treated with 1× tricaine from 30 hpf, did not show a significant increase in p-ERK levels normalised to total ERK levels (Fig. 4F,G), suggesting that the increase of Vegfa/Vegfr signalling in the trunk ISV of *svep1* morphants is not ubiquitous across the endothelium. Overall, these results suggest that the DLAV phenotype present in *svep1* morphants treated with tricaine is associated with a defect in tip/stalk cell specification in primary angiogenic sprouts, potentially through the modulation of Vegfa/Vegfr signalling.

To verify that the observed defect in tip cell specification is not secondary to the further reduction in blood flow speed observed in *svep1* loss-of-function embryos, we investigated tip cell identity in control embryos treated with 2× tricaine compared with 1× tricaine, as it results in a blood flow reduction greater than that observed in *svep1* loss-of-function embryos treated with 1× tricaine [451 µm/s±231.6, as presented by our laboratory in a recent paper (Geudens et al., 2019), versus 521 µm/s±190]. We found no differences between embryos treated with 1× and 2× tricaine (Fig. 4H,I), suggesting that the defect in tip cell specification is primarily a consequence of *svep1* loss-of-function in reduced flow condition.

### *svep1* loss-of-function and knockdown are rescued by *flt1* knockdown

To investigate the functional relevance of this Vegfa/Vegfr signalling increase in *svep1* morphants, we decided to modulate it *in vivo*, first by targeting Flt1 expression. In mice and zebrafish, Flt1 mainly functions as a decoy receptor with high affinity for Vegfa during development to modulate the activation of the Vegfa/Vegfr signalling pathway (Wild et al., 2017; Ito et al., 1998; Hiratsuka et al., 1998). Alternative splicing of *flt1* generates two isoforms: a membrane-bound form (mFlt1), and a soluble form (sflt1, an alternative spliced and secreted form of mFlt1) (Kendall and Thomas, 1993). Reports have shown that sFlt1 acts as a negative regulator of tip cell formation in the zebrafish trunk (Krueger et al., 2011).

**Fig. 5.**
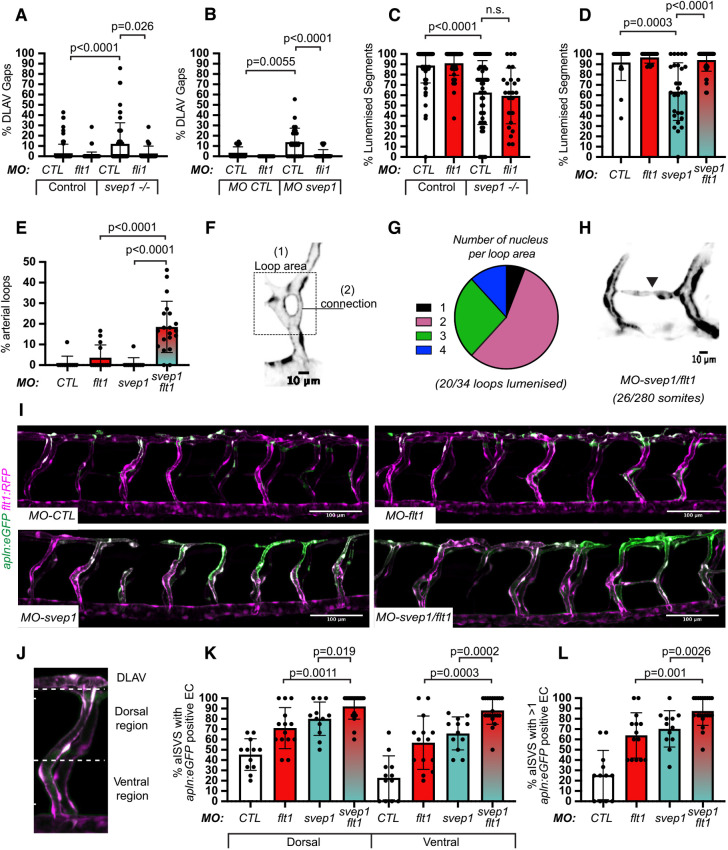
***svep1* loss-of-function and knockdown are rescued by *flt1* knockdown.** (A) Bilateral quantifications of the percentage of gaps in the DLAV at 48 hpf in controls and *svep1^hu4767−/−^* embryos injected with *MO-CTL* (5 ng) (*n*=45 and *n*=27, respectively) or *MO-flt1* (1 ng) (*n*=50 and *n*=12, respectively), and treated with 1× tricaine from 30 to 48 hpf (*N*=3). (B) Bilateral quantifications of the percentage of gaps in the DLAV at 48 hpf in *MO-CTL* (5 ng) (*n*=9), *MO-flt1* (1 ng) (*n*=7), *MO-svep1* (5 ng) (*n*=14) embryos (*N*=3), and treated with 1× tricaine from 30 to 48 hpf. (C) Bilateral quantifications of the percentage of lumenised segments in the DLAV at 48 hpf in controls and mutant *svep1^512−/−^* injected with *MO-CTL* (5 ng) (*n*=45 and *n*=27, respectively) or *MO-flt1* (1 ng) (*n*=50 and *n*=12, respectively) and treated with 1× tricaine from 30 to 48 hpf (*N*=3). (D) Bilateral quantifications of the percentage of lumenised segments in the DLAV at 48 hpf in *MO-CTL* (5 ng) (*n*=9), *MO-flt1* (1 ng) (*n*=7), *MO-svep1* (5 ng) (*n*=14) and *MO-flt1* (1 ng)/*MO-svep1* (5 ng) (*n*=25) embryos (*N*=3). (E) Bilateral quantifications of the percentage of aISV loops at 48 hpf in *MO-CTL* (5 ng) (*n*=11), *MO-flt1* (1 ng) (*n*=14), *MO-svep1* (5 ng) (*n*=11) and *MO-flt1* (1 ng)/*MO-svep1* (5 ng) (*n*=20) embryos (*N*=3). (F) Representative image of an arterial aISV loop in *MO-svep1* (5 ng)/*MO-flt1* (1 ng) *Tg(-0.8flt1:RFP)^hu3333^* embryos at 48 hpf, treated with 1× (0.014%) tricaine from 30 to 48 hpf. (G) Quantification of number of nuclei per loop area (see Fig. 4F) at 48 hpf in *MO-svep1* (5 ng)/*MO-flt1* (1 ng) embryos at 48 hpf, treated with 1× (0.014%) tricaine from 30 to 48 hpf (*n*=34 loops counted: 2, 19, 9 and 4 loops had 1, 2, 3 or 4 nuclei per loop area, respectively; 20/34 loops were lumenised) (*N*=3). (H) Representative image of an aISV-to-aISV connection in the region of the horizontal myoseptum at 48 hpf in in *MO-svep1* (5 ng)/*MO-flt1* (1 ng) *Tg(-0.8flt1:RFP)^hu3333^* embryos at 48 hpf, treated with 1× (0.014%) tricaine from 30 to 48 hpf (*n*=20 fish, 26 connections visible out of 280 somites, 8/26 connections were lumenised) (*N*=3). (I) Maximum intensity projection of a representative *TgBAC(apln:eGFP)^bns157^, Tg(-0.8flt1:RFP)^hu5333^* morphant embryo at 48 hpf. The panels show the GFP signal volume masked by the RFP signal to limit detection to the endothelium in *MO-CTL* (5 ng), *MO-flt1* (1 ng), *MO-svep1* (5 ng) and *MO-svep1* (5 ng)/*MO-flt1* (1 ng) embryos treated with 1× (0.014%) tricaine from 30 to 48 hpf. (J) Maximum intensity projection of a *MO-svep1* (5 ng)/*MO-flt1* (1 ng) aISV at 48 hpf, highlighting the ventral and dorsal region used for further quantifications in K. (K) Quantification of the percentage of aISVs with *apln:eGFP*-positive endothelial cells in the dorsal and ventral regions in 48 hpf *MO-CTL* (5 ng) (*n*=12), *MO-flt1* (1 ng) (*n*=14), *MO-svep1* (5 ng) (*n*=12) and *MO-svep1*(5 ng)/*MO-flt1* (1 ng) (*n*=20) morphant embryos treated with 1× (0.014%) tricaine from 30 to 48 hpf (*N*=3). (L) Quantification of the percentage of aISVs with more than one *apln:eGFP*-positive endothelial cell in 48 hpf *MO-CTL* (5 ng) (*n*=12), *MO-flt1* (1 ng) (*n*=14), *MO-svep1* (5 ng) (*n*=12) and *MO-svep1* (5 ng)/*MO-flt1* (1 ng) (*n*=20) morphant embryos treated with 1× (0.014%) tricaine from 30 to 48 hpf (*N*=3). Data are mean±s.d. Mann–Whitney test. Scale bars: 10 μm (H); 100 μm (I).

To reduce *flt1* expression, we used a morpholino targeting both mFlt1 and sFlt1 expression. Similar to previous observations (Wild et al., 2017), *flt1* morphants do not exhibit any DLAV defects at 48 hpf when treated with tricaine from 30 to 48 hpf. However, in *svep1* mutants and morphants, knockdown of *flt1* expression led to a rescue of DLAV formation defects (Fig. 5A,B). Interestingly, *flt1* knockdown rescued the DLAV segment lumenisation phenotype only in *svep1* morphants but not in mutant embryos (Fig. 5C,D), suggesting potential differences in the expressivity of the *flt1* knockdown in *svep1* mutant and morphants.

In addition to the DLAV rescue, *flt1/svep1* double knockdown led to the formation of aberrant arterial loops in aISVs (18.5%±12.4 versus 0.8%±2.7 in *MO-svep1* and 3.6%±6.1 in *MO-flt1* only) (Fig. 5E-G). The majority of these arterial loops were lumenised and comprised more than one endothelial cell (>1 cell/loop: 94.1% in *n*=34 loops). In 9.3% of somites (26/280) we also observed abnormal aISV-to-aISV connections (Fig. 5H), which were never seen in control embryos. Thus, the rescue of connectivity at the DLAV level through *flt1* knockdown produced an excess connectivity at aberrant locations.

Importantly, *flt1* morphants treated with 2× tricaine from 30 to 48 hpf exhibited aberrant arterial loops in only 2.4%±4.1 aISVs, whereas control morphants under the same treatment never exhibited any (*N*=3, *n*=21 *MO-flt1* 1 ng, *n*=24 *MO-CTL* 1 ng). In both conditions, we could not see any abnormal aISV-to-aISV connections. These results suggest that *svep1* loss-of-function, rather than reduced blood flow, is the principal driver for the excess connectivity observed with concomitant *flt1* knockdown.

In search for the underlying cause of hyperconnectivity, we next investigated tip cell specification. In embryos treated with 1× tricaine from 30 to 48 hpf, *flt1* knockdown led to an expansion and increase of the total number of *apln+* endothelial cells in aISVs, despite no significant DLAV formation defects (Fig. 5I-K). *svep1/flt1* double morphants exhibit significantly more *apln+* endothelial cells in the dorsal and ventral part of aISVs, and more *apln+* cells per aISV than both *svep1* and *flt1* morphants alone (more than one *apln+* cell in 87.6%±13.9 ISVs versus 70.3%±17.7 in *svep1* only morphants and 63.9±21.9 in *flt1* only morphants) (Fig. 5L).

These results suggest that the expansion of the number of tip cells in aISVs is not the driver of DLAV anastomosis defects, but potentially the cause for aberrant aISV-to-aISV connections.

### Vegfa/Vegfr signalling is necessary for ISV lumenisation maintenance and DLAV formation

Vegfa/Vegfr signalling regulates primary angiogenic sprouting in the developing zebrafish trunk. Inhibition of Vegfr tyrosine kinase activation between 18 and 20 hpf results in the absence of angiogenic sprouting from the DA (Fish et al., 2017). However, to our knowledge, there exist no reports for the role of active Vegfa/Vegfr signalling in the initial formation and lumenisation of the DLAV (30-48 hpf). As a polarised increase in Vegfa/Vegfr signalling is necessary to establish tip and stalk cell identity in the growing sprouts (Shin et al., 2016) and a local increase in VEGFA signalling is essential for the establishment of stable connections between vascular sprouts *in vivo* and *in vitro* (Nesmith et al., 2017), we decided to investigate the effect of a general reduction of this signalling pathway on the formation of a lumenised DLAV. For this purpose, we used the VEGFR2 inhibitor ZM323881, a tyrosine-kinase inhibitor that reduces Vegfa/Vegfr signalling (Whittles et al., 2002), confirmed by downregulation of p-ERK in fluorescence-activated cell sorting (FACS)-sorted endothelial cells from embryos treated with 50 nM ZM323881 and 1× tricaine from 30 to 48 hpf (Fig. S3A,B). Consistent with its function, ZM323881-mediated downregulation of Vegfa/Vegfr signalling can be partially rescued with downregulation of *flt1* expression (Fig. S3E,F). Embryos treated with 1× tricaine and 100 nM or 150 nM ZM323881 from 30 to 48 hpf exhibited significant DLAV defects (Fig. S3C,D).

### Vegfa/Vegfr signalling inhibition exacerbates *svep1* loss-of-function DLAV phenotype in reduced flow conditions

As the DLAV phenotype in *svep1* morphants correlates with an increase in Vegfa/Vegfr signalling and tip cell numbers (Fig. 4), and because an expansion of tip cell numbers within aISVs does not result in ipsilateral anastomosis defect (Fig. 5), we decided to investigate whether the increased Vegfa/Vegfr signalling observed in *svep1* morphants is correlative/compensatory or causative for the DLAV defects observed under reduced flow conditions.

**Fig. 6.**
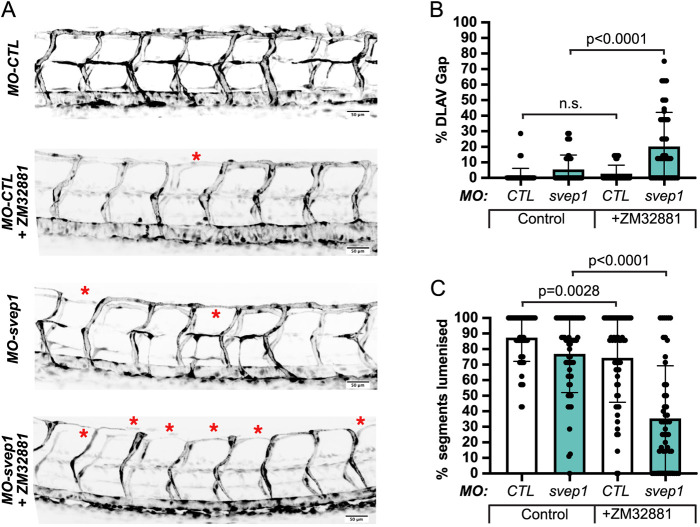
**Vegfa/Vegfr signalling is necessary for ISV lumenisation maintenance and DLAV formation.** (A) Maximum intensity projections at 48 hpf of the trunk of *MO-CTL* (5 ng) and *MO-svep1* (5 ng), *Tg(fli1a:eGFP)^y7^* embryos treated with 1× (0.014%) tricaine, with or without 50 ng ZM32881. Red asterisks indicate gaps in the DLAV. (B) Bilateral quantifications of the percentage of gaps in the DLAV at 48 hpf in *MO-CTL* (5 ng) [*n*=29 (0 nM ZM32881), *n*=28 (50 nM ZM32881)], *MO-svep1* (5 ng) [*n*=29 (0 nM ZM32881), *n*=26 (50 nM ZM32881)] embryos treated with 1× (0.014%) tricaine and 0 nM or 50 nM ZM32881 from 30 to 48 hpf (*N*=3). (C) Bilateral quantifications of the percentage of lumenised segments in the DLAV at 48 hpf in *MO-CTL* (5 ng) [*n*=29 (0 nM ZM32881), *n*=28 (50 nM ZM32881)], *MO-svep1* (5 ng) [*n*=29 (0 nM ZM32881), *n*=26 (50 nM ZM32881)] embryos treated with 1× (0.014%) tricaine and 0 nM or 50 nM ZM32881 from 30 to 48 hpf (*N*=3). Data are mean±s.d. Mann–Whitney test. n.s., not significant. Scale bars: 50 μm (A).

We took advantage of the variability in expressivity of the DLAV phenotype observed in different reporter lines (Fig. S1) to select one presenting with a limited DLAV phenotype when injected with *svep1* morpholino and treated with 1× tricaine from 30 to 48 hpf. In this context, we found that concomitant treatment with 50 nM ZM323881 results in a significant increase in the number of gaps in the DLAV (20.3%±21.8 versus 5.4%±9.3) and in a reduction of the number of lumenised DLAV segments (35.4±33.9 versus 76.9±24.9) (Fig. 6A-C; Fig. S4). Similarly, we found that treatment with the commonly used VEGFR signalling inhibitor SU5416 (Covassin et al., 2006; Zygmunt et al., 2012; Fong et al., 1999; De Angelis et al., 2017) also exacerbates the DLAV phenotype in *svep1* morphants treated with 1× tricaine from 30 to 48 hpf (Fig. S5A-C).

**Fig. 7.**
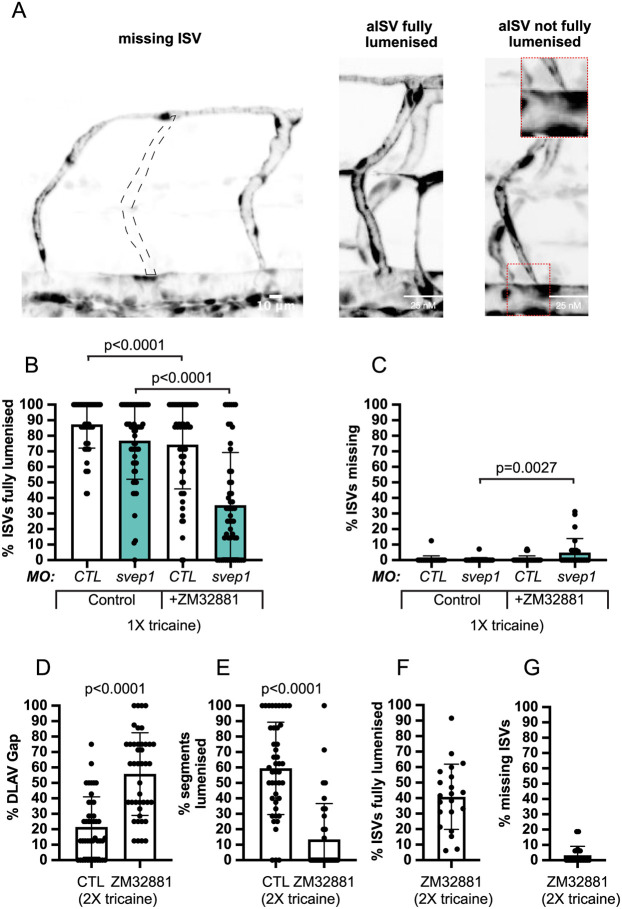
**Vegfa/Vegfr signalling is necessary for ISV lumenisation maintenance and DLAV formation.** (A) Representative images of missing ISV, fully lumenised ISV and not fully lumenised ISV at 48 hpf. Quantifications of these phenotypes are presented in B and C. Dashed line indicates expected location of missing ISV. Inset on right is magnification of boxed area. (B) Bilateral quantifications of the percentage of missing ISVs in the trunk of 48 hpf *MO-CTL* (5 ng) [*n*=29 (0 nM ZM32881), *n*=28 (50 nM ZM32881)], *MO-svep1* (5 ng) [*n*=29 (0 nM ZM32881), *n*=26 (50 nM ZM32881)] embryos treated with 1× (0.014%) tricaine and 0 nM or 50 nM ZM32881 from 30 to 48 hpf (*N*=3). (C) Bilateral quantifications of the percentage of ISVs lumenised dorsally to ventrally in the trunk of 48 hpf *MO-CTL* (5 ng) [*n*=29 (0 nM ZM32881), *n*=28 (50 nM ZM32881)], *MO-svep1* (5 ng) [*n*=29 (0 nM ZM32881), *n*=26 (50 nM ZM32881)] embryos treated with 1× (0.014%) tricaine and 0 or 50 nM ZM32881 from 30 to 48 hpf (*N*=3). (D) Bilateral quantifications of the percentage of gaps in the DLAV at 48 hpf in embryos treated with 2× (0.028%) tricaine and 0 (*n*=22) or 50 nM (*n*=21) ZM32881 from 30 to 48 hpf (*N*=3). (E) Bilateral quantifications of the percentage of lumenised segments in the DLAV at 48 hpf in embryos treated with 2× (0.028%) tricaine and 0 nM (*n*=22) or 50 nM (*n*=21) ZM32881 from 30 to 48 hpf (*N*=3). (F) Bilateral quantifications of the percentage of ISVs lumenised dorsally to ventrally in the trunk of 48 hpf in embryos treated with 2× (0.028%) tricaine and 50 nM (*n*=21) ZM32881 from 30 to 48 hpf (*N*=3). (G) Bilateral quantifications of the percentage of missing ISVs in the trunk of 48 hpf in embryos treated with 2× (0.028%) tricaine and 50 nM (*n*=21) ZM32881 from 30 to 48 hpf (*N*=3). Data are mean±s.d. Mann–Whitney test.

In addition, Vegfr inhibition impaired ISV lumenisation at 48 hpf (Fig. 7A), suggesting that beyond its importance in the ipsilateral anastomosis of aISVs and DLAV lumenisation, Vegfa/Vegfr signalling is involved in the maintenance of aISV lumenisation status. Also *svep1* morphants exhibited a mild but significant reduction in ISV lumenisation that is increased by treatment with 50 nM ZM323881 (26.9%±22.6 versus 80.3%±16.5) (Fig. 7B). Furthermore, we found that concomitant *svep1* knockdown and Vegfa/Vegfr signalling inhibition led to the emergence of embryos with a significant number of ISVs missing at 48 hpf (4.8%±9 versus 0.2%±1.3) following treatment with ZM323881 and 1× tricaine from 30 to 48 hpf (Fig. 7C). Finally, we found that in embryos treated with 50 nM ZM323881 and 2× tricaine from 30 to 48 hpf, 40.9%±21 of ISVs were lumenised (compared with 26.9%±22.6 in mild *svep1* morphants treated with 1× tricaine and 50 nM Zm323881) and 3.3%±5.8 were missing at 48 hpf (compared with 26.9%±22.6 in mild *svep1* morphants treated with 1× tricaine and 50 nM Zm323881), suggesting that reduced *svep1* and reduced blood flow both contribute to the emergence of a vascular phenotype in the context of Vegfa/Vegfr signalling inhibition (Fig. 7D-G).

Overall, these results suggest that the increase in Vegfa/Vegfr signalling observed in *svep1* morphants under reduced flow conditions is not causative for the failure of ipsilateral aISVs to anastomose after 30 hpf, but potentially represents a compensatory effect. This is supported by the finding that Svep1 and Vegfa/Vegfr signalling appear to act synergistically to maintain vessel lumenisation and stability under low flow conditions.

## DISCUSSION

The question of how vessels anastomose remains incompletely understood, given the complexities of endogenous and exogenous signals driving vascular remodelling and development in parallel and sometimes synergistic ways (Hogan and Schulte-Merker, 2017). Careful analysis of early-stage angiogenesis has shed light on the temporal and morphological dynamics underlying this process (Betz et al., 2016; Herwig et al., 2011; Lenard et al., 2013). Following the establishment of a stable connection between neighbouring tip cells, supported by the local deposition of adherens junction proteins, such as VE-cadherin, F-actin and ZO-1 (Lenard et al., 2013; Phng et al., 2013), ISV connections become progressively lumenised. Lumenisation and stabilisation of ISV connections is thought to occur either through a flow-dependent transcellular hollowing of connecting tip cells (Type I anastomosis) (Lenard et al., 2013; Gebala et al., 2016) or through a flow-independent process involving the coalescence of isolated luminal pockets into a single luminal space that will subsequently be perfused (Type II anastomosis) (Herwig et al., 2011). However, comparatively little is known about the molecular pathways leading to the formation and stabilisation of nascent anastomotic connections.

Here, we identified blood flow and Svep1 as regulators of vessel anastomosis in the developing zebrafish vasculature. Both appear to play a role in the stabilisation of nascent anastomotic connections between neighbouring vessels. Under reduced flow conditions, *svep1* knockdown or loss-of-function resulted in reduced anastomosis of ipsilateral ISVs and defective formation of a lumenised DLAV at 48 hpf. We found that endothelial cells in *svep1* morphants displayed increased Vegfa/Vegfr signalling, which is consistent with a concomitant increase in Apelin-positive cells in ISVs. Neither of these events, however, appear to be causative, as the DLAV defect can be rescued with *flt1* knockdown and is exacerbated by Vegfa/Vegfr signalling inhibition. Interestingly, although inhibition of blood flow speed below that exhibited in a context of *svep1* loss-of-function and 1× tricaine treatment does similarly result in visible anastomosis defect in the DLAV region, these are not associated with a significant increase of Apelin-positive cells in the trunk ISV. Therefore the expansion of the number of Apelin-positive tip cells in aISVs is unlikely to be a consequence of the reduced anastomosis or lumenisation, and also not the causal driver of anastomosis defects. Rather, our data suggest that the increased number of Apelin-positive cells is related to the increased Vegfa/Vegfr signalling, as this is also seen in the *flt1* knockdown situation, and even exacerbated when both *svep1* and *flt1* are knocked down. Thus the increase in Vegfa/Vegfr signalling and Apelin-positive tip cell numbers in the absence of *svep1* are likely compensatory, given that further increases of both in the flt1 knockdown condition can rescue the DLAV defects, whereas Vegfr inhibition exacerbates the loss of DLAV connections. In previous work in the mouse retina, Nesmith and colleagues have shown that endothelial cells presenting with reduced Flt1 expression were more likely to form stable connections with approaching sprouts (Nesmith et al., 2017). They also clarified that reduced mFlt1 expression is what influences the bias towards stable connections, suggesting a cell-autonomous regulation of tip cell anastomosis. Finally, they remarked that sprouts with reduced Flt1 expression exhibit reduced retraction of nascent connections with adjacent sprouts, proposing that this might lead to an increase in the number of suboptimal but stabilised new vascular connections. We noticed a significant increase of aberrant vascular loops within ISVs, which can functionally be considered suboptimal or redundant for perfusion of tissues, in embryos with reduced *svep1* and *flt1* expression compared with *flt1* knockdown alone. On its own, this result suggests that the role of Svep1 in vascular development might occur through the modulation of Flt1 activity *in vivo*. As the function of Flt1 in developmental angiogenesis appears to be strictly limited to its role as a decoy receptor for VEGFA (Hiratsuka et al., 1998; Fong et al., 1995), we can speculate that Svep1 might support/enhance Flt1 decoy ability. However, under reduced flow conditions, although *flt1* knockdown enhances the formation of stable connections between ipsilateral neighbouring sprouts, we found that *svep1* knockdown alone led to a significant reduction in the stability of these connections. In addition, we found that inhibition of Vegfa/Vegfr signalling led to a DLAV phenotype comparable with that observed in *svep1* mutant and morphants, in which Vegfa/Vegfr signalling levels are significantly upregulated, and that Vegfa/Vegfr inhibition in *svep1* morphants led to a strong increase of anastomosis defects. Taken together, these results reinforce the conclusion that the observed increase in Vegfa/Vegfr signalling following *svep1* knockdown is compensatory in nature but insufficient to stabilise new connections in the absence of flow.

The importance of blood flow inhibition in the emergence of the DLAV phenotype might suggest a potential investigative avenue. In embryos with reduced blood flow and concomitant reduced Svep1 levels or reduced Vegfa/Vegfr signalling, we observed a significant reduction in ISV lumenisation. This suggests that in this context, a significant proportion of ISVs might initiate anastomosis through flow-independent pathways (Type II anastomosis). We can speculate that blood flow plays a positive role in the stabilisation of Type II anastomosis between neighbouring ISVs, which would explain the DLAV formation defect observed in reduced (tricaine treatment) or abolished (*tnnt2a* morpholino) blood flow conditions. The significant increase in the DLAV phenotype observed in *svep1* loss-of-function and abolished flow conditions suggests a role of Svep1 in regulating vessel anastomosis in reduced flow conditions.

In addition, although blood flow reduction between 30 and 48 hpf is sufficient to induce anastomotic defects in the DLAV, this phenotype is significantly exacerbated with concomitant treatment with Vegfr signalling inhibitors, supporting the idea that both flow and Vegfa/Vegfr signalling may act synergistically to support stable vessel anastomosis.

Svep1 is a secreted ECM protein that has been reported to mediate cell-to-substrate adhesion *in vitro*, in an integrin α9β1-dependent manner (Sato-Nishiuchi et al., 2012). Although integrin α9 zebrafish mutants fail to display a vascular phenotype other than a defect in lymphatic valve formation (Shin et al., 2019), interaction with another member of the integrin family could prove more relevant. For example, Integrin β1b appears to be important for the formation of the DLAV in zebrafish embryos (Iida et al., 2018). Future efforts in identifying interaction partners for Svep1 will further enhance our understanding of the molecular pathways regulating vessel anastomosis. Imaging of the *svep1* reporter line *Tg(svep1:Gal4FF;UAS:GFP)* at 48 hpf showed strong GFP expression in dorsal epithelial cells, above the neural tube, and in individual neurons of the neural tube in close proximity to the anastomotic bridges forming between adjacent ISVs. We can speculate that neuronal expression of Svep1 is what locally regulates vessel anastomosis, in a non-cell-autonomous manner. In addition, Svep1 is a multi-domain protein, and the importance of each domain could be functionally tested in reduced flow conditions, by the generation of zebrafish lines expressing selective truncated forms of Svep1. Finally, the significant differences observed in *svep1* loss-of-function phenotype expressivity between zebrafish lines might offer an interesting avenue to decipher the compensatory mechanisms at play, and reveal new molecular pathways interacting with Svep1 in the regulation of vessel anastomosis. In the absence of clear structure-function details on Svep1 protein and knowledge of relevant molecular receptor interactions in endothelial cells, our mechanistic insights into how the non-endothelial expression of Svep1 supports vessel anastomosis remains very limited. Equally open remains the question of whether there is a threshold of blood flow that is crucial for stabilising nascent anastomotic connections and, if so, whether this is similar in different vascular beds.

Understanding the genetic regulation of phenotypic robustness in angiogenesis and its failure (Kasper et al., 2017) promises crucial insights into the mechanisms causing breakdown of vascular homeostasis in human disease. The search for genetic causes for vascular disease is particularly challenging due to multifactorial risk profiles and multigenic disease mechanisms. Interestingly, however, Svep1 is evolutionarily highly conserved, and genotyping efforts by the Myocardial Infarction Genetics and CARDIoGRAM Exome Consortia identified missense variants in Svep1 associated with coronary artery disease and elevated blood pressure (Myocardial Infarction Genetics and CARDIoGRAM Exome Consortia Investigators, 2016). Svep1 loss-of-function mutations were also associated with worse outcome in septic shock (Nakada et al., 2015). Although the underlying mechanisms remain to be investigated, it is noteworthy that disturbed endothelial responses to flow are a central mechanism in atherosclerosis, and that vessel rarefaction due to breakdown of connectivity increases peripheral resistance, and thus blood pressure. The link between Flt1 protein levels and reduced Vegfa/Vegfr signalling provides a well-known mechanism for vessel rarefaction and hypertension, for example in preeclampsia. Intriguingly, Svep1 mRNA is also strongly downregulated in cytotrophoblast populations in preeclampsia (Gormley et al., 2017). Future work will need to address whether our newly identified connection between blood flow and Svep1 function is involved in the pathomechanisms underlying vessel loss, coronary artery disease or other aspects of vascular dysfunction in humans.

## MATERIALS AND METHODS

### Zebrafish husbandry and transgenic lines

Zebrafish (*Danio rerio*) were raised and staged as previously described (Kimmel et al., 1995). The following transgenic lines were used: *Tg[fli1a:EGFP]^y1^* (Lawson and Weinstein, 2002) (labels all endothelial cells), *Tg[gata1a:dsRed]^sd2^* (Traver et al., 2003) (labels all erythrocytes), *Tg[-0.8flt1:RFP]^hu5333^* (Bussmann et al., 2010) (strongly labels arterial endothelial cells), *TgBAC(apln:eGFP)^bns157^* (Marín-Juez et al., 2019) (labels endothelial tip cells), *TgBAC[svep1:GAL4FF]^hu8885^;Tg(5xUAS:EGFP)^nkuasgfp1a^ Tg(svep1:Gal4FF;UAS:GFP)* (labels *svep1*-positive cells) (Karpanen et al., 2017), *Tg(kdrl:mcherry-CAAX)^y171^* and *TgBAC(flt4:Citrine)*. The *svep^hu4767^* mutant line has been described previously (Karpanen et al., 2017). For growing and breeding of transgenic lines we comply with regulations of the ethical commission animal science of MDC Berlin and with FELASA guidelines (Alestrom et al., 2019).

### Tricaine treatment

To slow down heart rate and blood flow during DLAV formation, embryos were treated with 0.007%, 0.014% (1×) or 0.028% (2×) tricaine (MS-222, Sigma-Aldrich) between 30 and 48 hpf, as indicated in the figure legends. For Fig. 3A,B and Fig. S3C 1× tricaine was 0.0168%.

### Morpholino knockdown

Morpholinos against *svep1* (5 ng), *flt1* (1 ng), *gata1* (8 ng) and *tnnt2a* (4 ng) were used as previously described (Zygmunt et al., 2012; Morooka et al., 2017; Wild et al., 2017; Amigo et al., 2009) and injected in the yolk of zebrafish embryos at the one-cell stage.

### mRNA injections

To generate mRNA for injections, pmtb-t7-alpha-bungarotoxin (gift from Sean Megason, Harvard Medical School, MA, USA) (Addgene plasmid #69542; RRID:Addgene_69542) (Swinburne et al., 2015) was digested with EcoRV and mRNA transcribed using HiScribe T7 ARCA mRNA Kit (New England Biolabs). Control mRNA (GFP) was translated from XbaI-digested pCS2+EGFP using the mMESSAGE mMACHINE SP6 Transcription kit (Invitrogen), and 50 pg of mRNA was injected into one- to two-cell-stage embryos as described.

### *In situ* hybridisation

A synthetic, double stranded DNA fragment (Eurofins) with the complete coding sequence from *Danio rerio klf2a* mRNA (Genbank accession number: NM_131856) was inserted into EcoRV-digested pISceI using NEBuilder HiFi DNA Assembly (New England Biolabs). The plasmid was digested with BamHI (anti-sense) or XhoI (sense) and probes transcribed using 20 U/μl T7 (anti-sense) or Sp6 (sense) polymerase (Promega) with digoxigenin labelling mix. Then, 48 hpf embryos were treated with 0.02 mg/μl Proteinase K for 30 min. The *in situ* hybridisations were performed as previously described (Thisse and Thisse, 2008; Nuesslein-Volhard, 2002). Embryos were dehydrated in methanol and mounted in glycerol for imaging.

### Statistical analysis

All quantifications were performed in the trunk region of zebrafish embryos, across 7-9 somites. *N* refers to experiments, *n* refers to embryos. Statistical analysis was performed with Mann–Whitney *U*-tests, unless indicated otherwise. No statistical method was used to predetermine sample size. Data represent mean±s.d. of representative experiments (except when indicated otherwise). Statistical tests were conducted using Prism (GraphPad) software. Adequate tests were chosen according to the data to fulfil test assumptions. Sample sizes, number of repeat experiments, performed tests and *P*-values are indicated per experiment.

Zebrafish embryos were selected based on the following pre-established criteria: normal morphology, beating heart and presence of circulating red blood cells. The experiments were not randomised. For every experiment treated and control embryos were derived from the same egg lay. The investigators were not blinded to allocation during experiments and outcome assessment.

### Live imaging

Embryos were anaesthetised in 0.014% tricaine (MS-222, Sigma-Aldrich), mounted in a 35 mm glass-bottom Petri dish (0.17 mm, MatTek) using 0.6-1% reduced melting point agarose (Sigma-Aldrich) containing 0.014% tricaine, and bathed in E3 media containing 0.014% tricaine and 0.003% *N*-phenylthiourea (PTU; Sigma-Aldrich). Time-lapse imaging was performed using an upright 3i spinning-disc confocal using a Zeiss Plan-Apochromat, 20×, 40×/1.0 NA water-dipping objective. Image processing was performed using Fiji software (Schindelin et al., 2012).

### Isolation of endothelial cells

We dechorionated 48 hpf *Tg[fli1a: nEGFP]^y7^* and *Tg[fli1a: EGFP]^y1^* crossed to *Tg[gata1:dsRed]* embryos using a solution of 1 mg/ml Pronase (Sigma-Aldrich) on an orbital shaker for 10 min at room temperature. Up to 250 dechorionated embryos per conditions were anaesthetised with 1× (0.014%) tricaine and transferred to a 1.5 ml Eppendorf with 1 ml calcium-free Ringer solution (116 mM NaCl, 2.9 mM KCl, 5 mM HEPES pH 7.2) to remove the yolks. After pipetting gently up and down with a 1 ml tip, the embryos were centrifuged at 2000 rpm (450 ***g***) for 5 min at 4°C. The supernatant was removed, and the procedure repeated until all the yolks were removed and the solution clear. The calcium-free ringer solution was replaced with 1 ml of protease solution [72 µg/ml Liberase DH research grade (Merck/Sigma), 0.4 U/ml DNaseI (Invitrogen)]. The embryos were incubated at 28.5°C on an orbital shaker for 20 min, pipetting up and down with a 200 µl tip every 3 min, to form a homogenous solution of cells. The dissociation process was stopped by placing the embryos on ice and adding 2 µl CaCl_2_ and 0.5 µl fetal bovine serum (FBS) per ml. The cell suspension was centrifuged at 2000 rpm (450 ***g***) for 5 min at 4°C. The supernatant was discarded, and the cells resuspended in sorting solution (2 mM EDTA, 0.4 U/ml DNaseI, 0.5% FBS in DPBS). The solution was passed through a 40 µm strainer inside a 50 ml falcon tube, previously washed with 500 µl sorting solution. Following filtration, 500 µl sorting solution was added to the strainer. The filtered solution was centrifuged at 2000 rpm (450 ***g***) for 5 min at 4°C. The supernatant was removed, and the cell resuspended in 700 µl sorting solution. The cell suspension was then loaded onto an ARIA III FACsorter (BD Biosciences). Using *Tg[gata1:dsRED]* only embryos for gating, we specifically sorted GFP+, dsRED− cells to remove red blood cells with active *fli1a* promoter activation at that developmental stage. The cells were then centrifuged, all the supernatant removed, and cells stored immediately at −80°C until protein extraction.

### Protein extraction

Sorted embryos were treated with 40 µl Lysis Buffer (1 ml 1 M Tris-HCl, 0.4 ml 0.5 M EDTA, 8.75 ml 10% Brij 96, 1.25 ml 10% NP-40, made up to 100 ml with dH_2_O) and 0.4 µl Protease Inhibitor cocktail (Thermo Fisher Scientific). The samples were homogenised with a pestle and centrifuged at 13,000 rpm (16,000 ***g***) for 15 min at 4°C. The protein supernatant was collected, and protein concentration assessed using a BCA protein assay kit (Thermo Fisher Scientific).

### Western blot

We diluted 20-50 µg of the protein lysates (equal amount for each condition to compare) in 18.7 µl water and 6.3 µl loading buffer (25 µl total volume) and heated for 5 min at 95°C to denaturise the proteins. The samples were loaded and run alongside a 10 µl ladder marker (Novex Sharp Pre-Stained, Thermo Fisher Scientific) for 1 h at 150 V and subsequently transferred onto previously MeOH-activated polyvinylidene fluoride membranes. Membranes were blocked with blocking solution [5% nonfat dry milk in 50 mg/ml Tris-buffered saline, 0.1% Tween 20 (TBS-T)] for 1.5 h at room temperature and then incubated with primary antibody against p-ERK [1:250, (Erk1/2) (Thr202/Tyr204), #9101, Cell Signaling Technology) overnight at 4°C. After incubation with primary antibody, the membranes were washed four times in 50 mg/ml TBS-T and then incubated with secondary antibodies (1:4000, horseradish peroxidase-conjugated donkey anti-rabbit, Sigma-Aldrich, GENA934-1ML) and washed three times with 50 mg/ml TBS-T. Immunodetection was performed using a chemiluminescence kit (1 ml SuperSignal West Dura; Pierce), and bands were developed using the Las-4000 imaging system. After initial immunodetection, membranes were stripped of antibodies using the Stripping kit (Thermo Fisher Scientific) at 56°C for 40 min and re-probed with anti-GFP antibody for 1 h (1:1000, Origene, R1091P). Band intensity was measured, using the histogram function on the Fiji software, with control and treated samples on the same blot (Schindelin et al., 2012).

### Blood flow and heart rate measurements

Embryos were anaesthetised in 0.014% tricaine (MS-222, Sigma-Aldrich) from 30 to 48 hpf before being mounted in a Petri dish using 1% low melting point agarose (Sigma-Aldrich) containing 0.014% (1×) tricaine, and bathed in Danieau's buffer containing (1×) tricaine respectively and 0.003% PTU, and imaged on an upright 3i spinning-disc confocal using a Zeiss Plan-Apochromat 20×/1.0 NA water-dipping objective with a frame interval of 10 ms. Kymographs were generated using the MultipleKymograph plugin in ImageJ to quantify heart rate over an 8 s period, synced to the beginning of a heartbeat (line width: 1).

To estimate instantaneous blood flow speed, we cropped images of the DA and measured average frame-to-frame translation of red blood cells using the Kuglin-Hines algorithm (Kuglin and Hines, 1975) for image phase-correlation. In brief, the phase correlation map between two adjacent frames was calculated by multiplying the Fast Fourier transform (FFt) of frame_i_ and a conjugate FFt of frame_i+1_. The inverse FFt of the phase correlation gives a correlation map with a peak offset from the centre by the relative shift between the frames. The position of the peak was determined by finding the local maximum in a Gaussian-filtered correlation map. The velocity data was smoothed with a moving average filter with a span of five frames. Analysis was performed in Matlab (Mathworks).

### Chemical treatment

Where indicated, embryos were treated with VEGFR inhibitors ZM323881 (Tocris Bioscience) or SU5416 (Sigma-Aldrich), from 30 to 48 hpf, in addition to 0.003% PTU and the indicated amount of tricaine.

## Supplementary Material

Supplementary information

Reviewer comments
