# Supplementary material for: Svep1 stabilises developmental vascular anastomosis in reduced flow conditions
Source: Development. 2022 Mar 25;149(6):dev199858. doi: 10.1242/dev.199858 (PMC8977097; doi:10.1242/dev.199858)
Supplement: Supplementary information [file develop-149-199858-s1.pdf]

Supplementary Figure 1

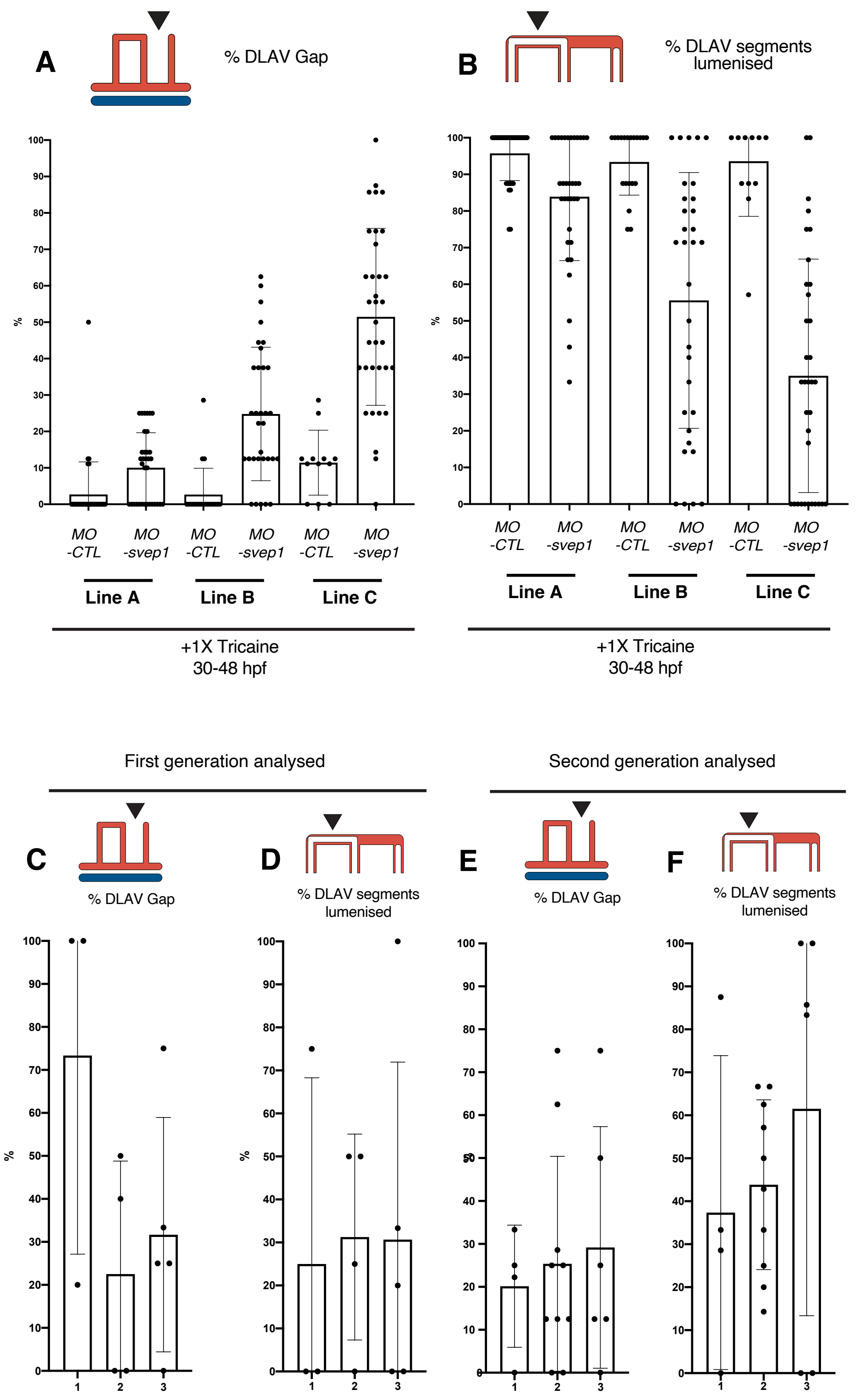

Development • Supplementary information

Fig. S1. (A) Bilateral quantifications of the percentage of gaps in the DLAV at 48 hpf in Line A: *Tg(fli1a:eGFP)y1* (N=3, n=18 *MO-CTL* 5ng, n=17 *MO-svep1* 5ng), Line B: *TgBAC(apln:eGFP); Tg(-0.8flt1:RFP)* (N=3, n=10 *MO-CTL* 5ng, n=16 *MO-svep1* 5ng), Line C: *TgBAC(flt1:YFP), Tg(kdrl:mCherry)* (N=3, n=6 *MO-CTL* 5ng, n=17 *MO-svep1* 5ng). The embryos were treated with 1X (0.014%) tricaine from 30 to 48 hpf.

(B) Bilateral quantifications of the percentage of lumenised segments in the DLAV at 48 hpf in Line A: *Tg(fli1a:eGFP)y1* (N=3, n=18 *MO-CTL* 5ng, n=17 *MO-svep1* 5ng), Line B: *TgBAC(apln:eGFP); Tg(-0.8flt1:RFP)* (N=3, n=10 *MO-CTL* 5ng, n=16 *MO-svep1* 5ng), Line C: *TgBAC(flt1:YFP), Tg(kdrl:mCherry)* (N=3, n=6 *MO-CTL* 5ng, n=17 *MO-svep1* 5ng). The embryos were treated with 1X (0.014%) tricaine from 30 to 48 hpf.

(C) Unilateral quantifications of the percentage of gaps in the DLAV at 48 hpf in *svep1*^{hu4767} homozygous mutants (n=3, 4, 5 embryos) treated with 1X tricaine (0.014%) from 30 to 48 hpf.

(D) Unilateral quantifications of the percentage of lumenised segments in the DLAV at 48 hpf in *svep1*^{hu4767} homozygous mutants (n=3, 4, 5 embryos) treated with 1X tricaine (0.014%) from 30 to 48 hpf.

(E) Bilateral quantifications of the percentage of gaps in the DLAV at 48 hpf in *svep1*^{hu4767} (n=2, 5, 3 embryos) treated with 1X tricaine (0.014%) from 30 to 48 hpf (N=3).

(F) Bilateral quantifications of the percentage of lumenised segments in the DLAV at 48 hpf in *svep1*^{hu4767} homozygous mutants (n=2, 5, 3 embryos) treated with 1X tricaine (0.014%) from 30 to 48 hpf (N=3).

Supplementary Figure 2

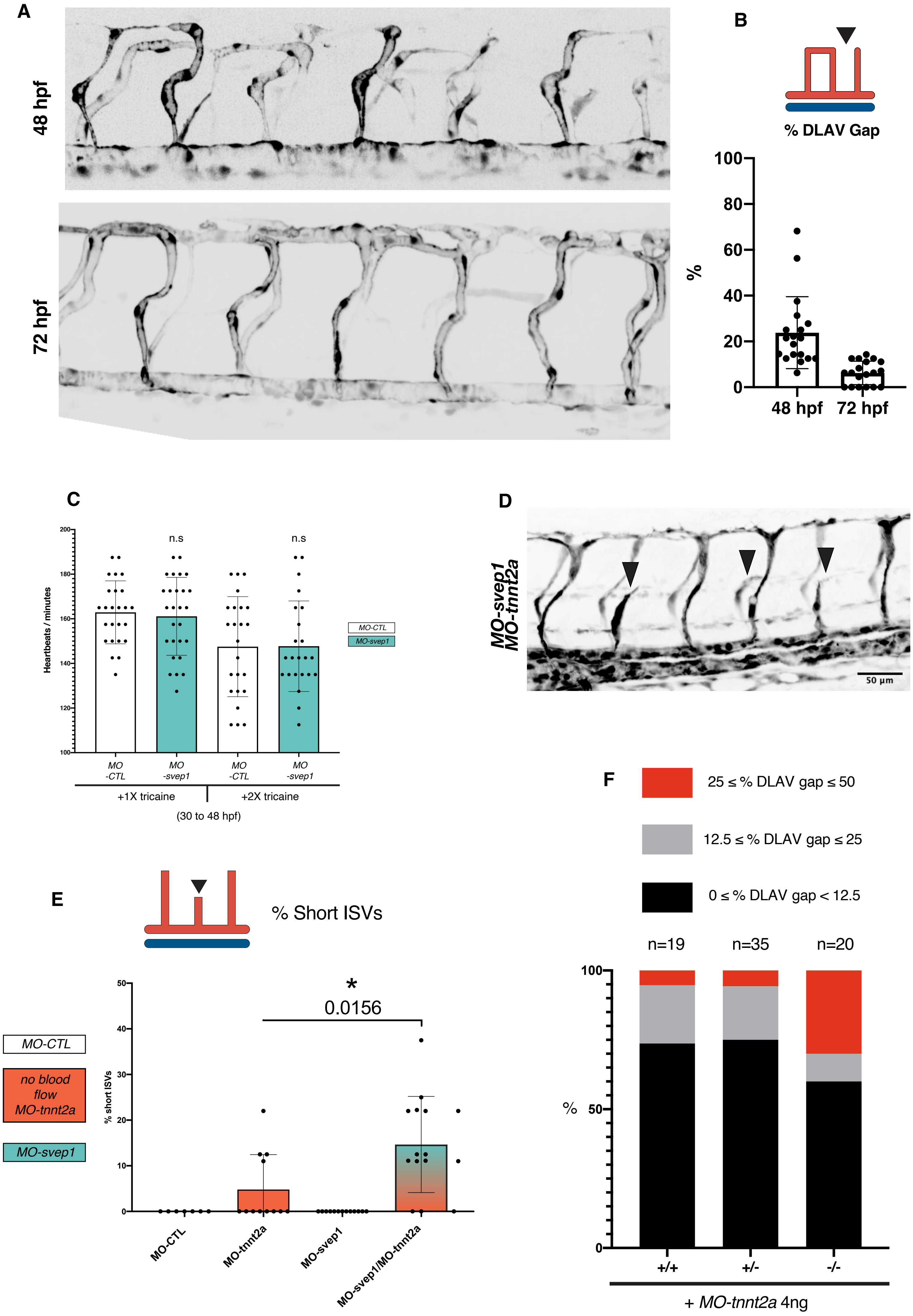

Development • Supplementary information

Fig. S2. (A) Representative image of a *Tg(-0.8flt1:RFP)*^{hu333}, svep1$^{hu4767\ -/-}$ embryo treated with 1X (0.014%) tricaine from 30 to 48 hpf and imaged at 48 and 72 hpf.

(B) Bilateral quantification of the percentage of gaps at 48 and 72 hpf in the DLAV of svep1$^{hu4767\ -/-}$ embryo treated with 1X (0.014%) tricaine from 30 to 48 hpf (n=19, N=3).

(C) Quantification of heart rate (heartbeats/minute) in the dorsal aorta of *Tg[gata1a:dsRed]*^{sd} MO-CTL (5ng) or MO-svep1 (5ng) embryos at 48 hpf, treated with 1X (0.014%)or 2X (0.028%) tricaine from 30 hpf. (N=4, n=25, 24 MO-CTL (1x, 2X), n=27, 23 MO-svep1 (1X, 2X)).

(D) Maximum intensity projection of zebrafish trunk in MO-svep1 (5ng)/Mo-tnnt2a (4ng) embryo at 48 hpf. black arrowheads indicate short ISVs.

(E) Bilateral quantification of the percentage of short ISVs in the trunk of 48 hpf MO-CTL (5ng)(n=7), MO-tnnt2a (4ng)(n=12), MO-svep1 (5ng)(n=13) and MO-svep1(5ng)/MO-tnnt2a (4ng)(n=15) embryos (N=3).

(F) Percentage of fish presenting with less than 12.5%, between 12.5 and 25% or above 25% gaps in the DLAV at 48 hpf in *svep1*$^{+/+}$ (n=19) , *svep1*$^{+/-}$ (n=35) or *svep* $^{-/-}$ (n=20) embryos injected with *MO-tnnt2a* (4ng) (N=4).

Development • Supplementary information

Supplementary Figure 3

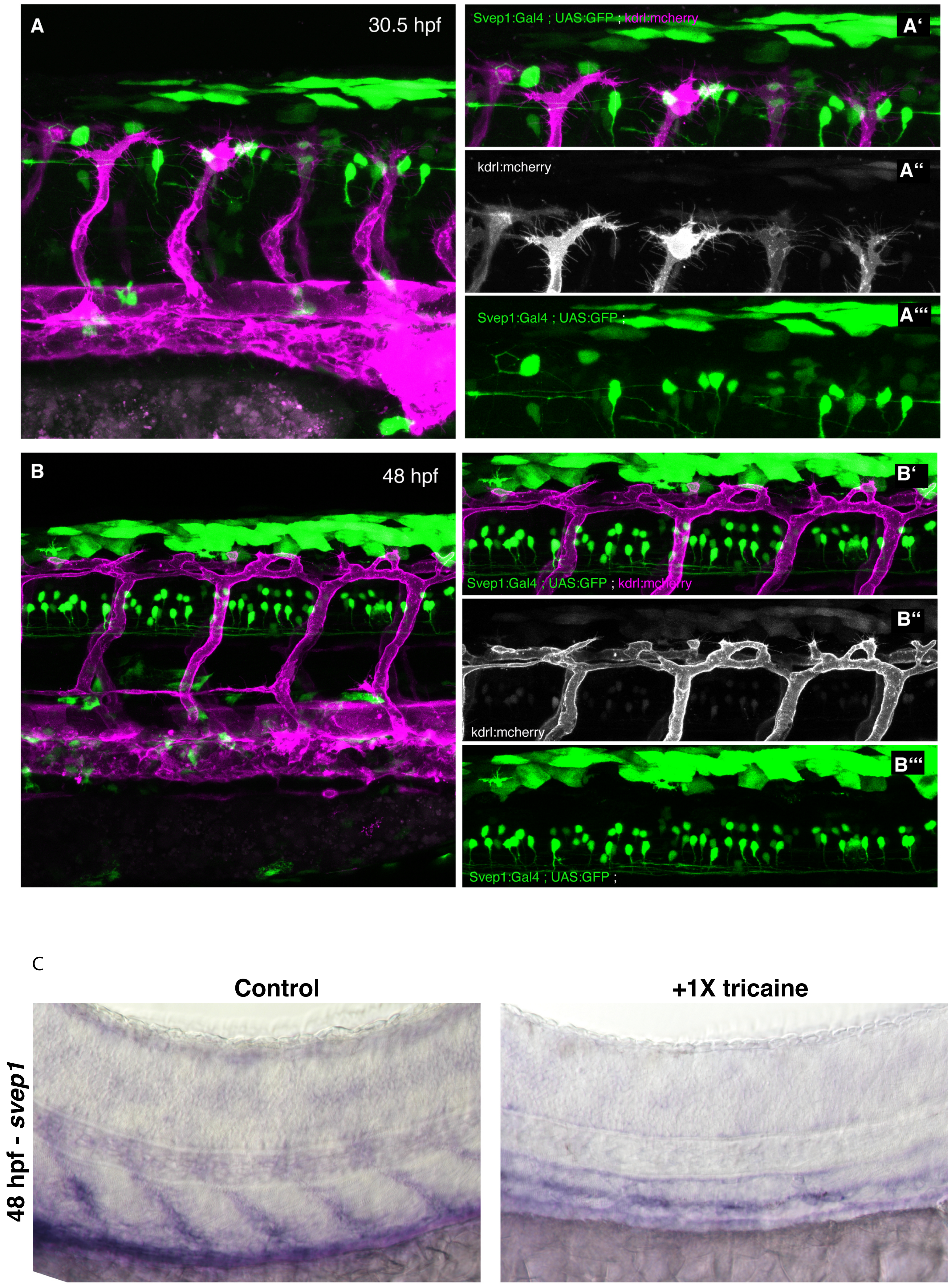

Development • Supplementary information

Fig. S3. (A) Representative images of 48 hpf *Tg(svep1:Gal4FF; UAS:eGFP); Tg(kdrl:mcherry-CAAX)^{y171}* in the trunk of embryos at 30.5 hpf.

(B) Representative images of 48 hpf *Tg(svep1:Gal4FF; UAS:eGFP); Tg(kdrl:mcherry-CAAX)^{y171}* in the trunk of embryos at 48 hpf.

(C) *In-situ svep1* endogenous expression in 48hpf embryos not treated (n=11) or treated with 1X (0.0168%) tricaine from 30 to 48 hpf (n=18).

Development • Supplementary information

Supplementary Figure 4

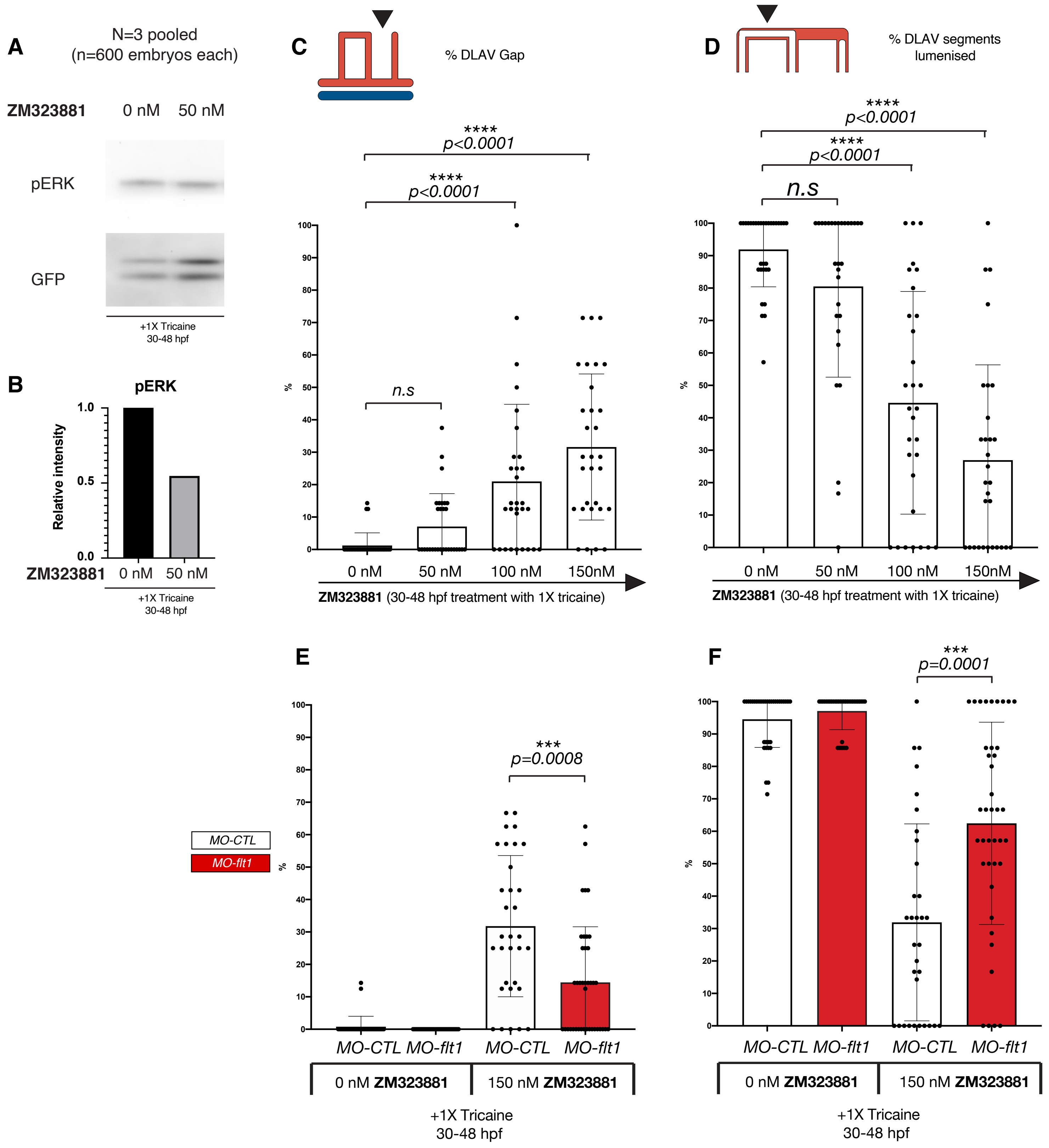

Fig. S4. (A) Representative image of p-ERK and GFP levels in FAC sorted endothelial cells from embryos treated with 1X (0.014%) tricaine from 30 to 48 hpf and

0nM (n=600 embryos – 290637 cells) or 50 nM (n=600 embryos, 300116 cells) ZM323881(N=3 experiments, pooled).

(B) Quantification of p-ERK in FAC sorted endothelial cells from embryos treated with 1X (0.014%) tricaine from 30 to 48 hpf and 0nM (n=600 embryos – 290637 cells) or 50 nM (n=600 embryos, 300116 cells) Expression levels were normalised to GFP levels (N=3 experiments, pooled).

(C) Bilateral quantifications of the percentage of gaps in the DLAV at 48 hpf in WT embryos treated with 1X (0.014%) tricaine and 0 (n=16), 50 (n=15), 100 (n=15) and 150 nM (n=15) ZM32881 from 30 to 48 hpf (N=3). Kruskall-Wallis Anova test.

(D) Bilateral quantifications of the percentage of lumenised segments in the DLAV at 48 hpf in WT embryos treated with 1X (0.014%) tricaine and 0 (n=16), 50 (n=15), 100 (n=15) and 150 nM (n=15) ZM32881 from 30 to 48 hpf (N=3). Kruskall-Wallis Anova test.

(E) Bilateral quantifications of the percentage of gaps in the DLAV at 48 hpf in *MO-CTL* (5 ng) (n=17 (0nM ZM32881), n=16 (150nM ZM32881)) *MO-flt1* (1ng) n=17 (0 nM ZM32881), n=20 (150nM ZM32881)) embryos treated with 1X (0.014%) tricaine and 0 or 50 nM ZM32881 from 30 to 48 hpf (N=4).

(F) Bilateral quantifications of the percentage of lumenized segments in the DLAV at 48 hpf in *MO-CTL* (5 ng) (n=17 (0nM ZM32881), n=16 (150nM ZM32881)) *MO-flt1* (1ng) (n=17 (0 nM ZM32881), n=20 (150nM ZM32881)) embryos treated with 1X (0.014%) tricaine and 0 or 50 nM ZM32881 from 30 to 48 hpf (N=4).

Development • Supplementary information

Supplementary Figure 5

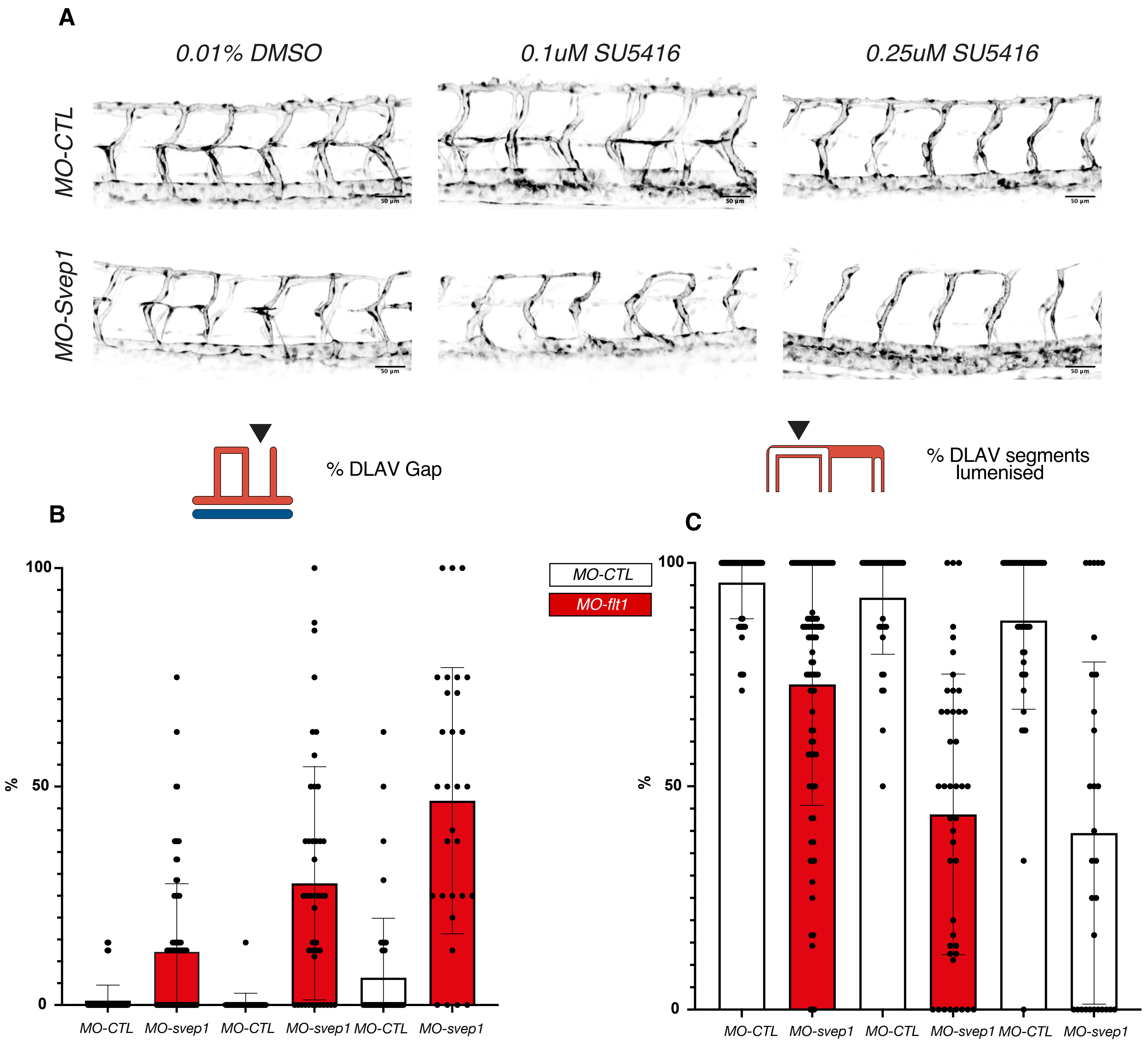

Development • Supplementary information

Fig. S5. A) Representative images of *Tg(fli1a:eGFP)*^{y1} embryos at 48 hpf, following treatment with 1X (0.014%) tricaine from 30 hpf, in combination with 0.01%DMSO, 0.1μM SU5416 or 0.25μM SU5416.

B) Bilateral quantifications of the percentage of gaps in the DLAV at 48 hpf in *MO-CTL* (5 ng) (n=80 (0.01% DMSO), n=38 (0.1μM SU5416), n=46 (0.25uM DMSO), n=30 (0.5μM SU5416)) *MO-svep1* (5ng) (n=88 (0.01% DMSO), n=44 (0.1μM SU5416), n=30 (0.25μM DMSO), n=22 (0.5μM SU5416)) embryos treated with 1X (0.014%) tricaine and 0.01% DMSO (N=6) or 0.1 (N=3), 0.25 (N=4), 0.5μM (N=4) SU5416 from 30 to 48 hpf.

C) Bilateral quantifications of the percentage of lumenised segments in the DLAV at 48 hpf in *MO-CTL* (5 ng) (n=80 (0.01% DMSO), n=38 (0.1μM SU5416), n=46 (0.25uM DMSO), n=30 (0.5μM SU5416)) *MO-svep1* (5ng) (n=88 (0.01%DMSO), n=44 (0.1μM SU5416), n=30 (0.25μM DMSO), n=22 (0.5μM SU5416)) embryos treated with 1X (0.014%) tricaine and 0.01% DMSO (N=6) or 0.1 (N=3), 0.25 (N=4), 0.5μM (N=4) SU5416 from 30 to 48 hpf.

Development • Supplementary information

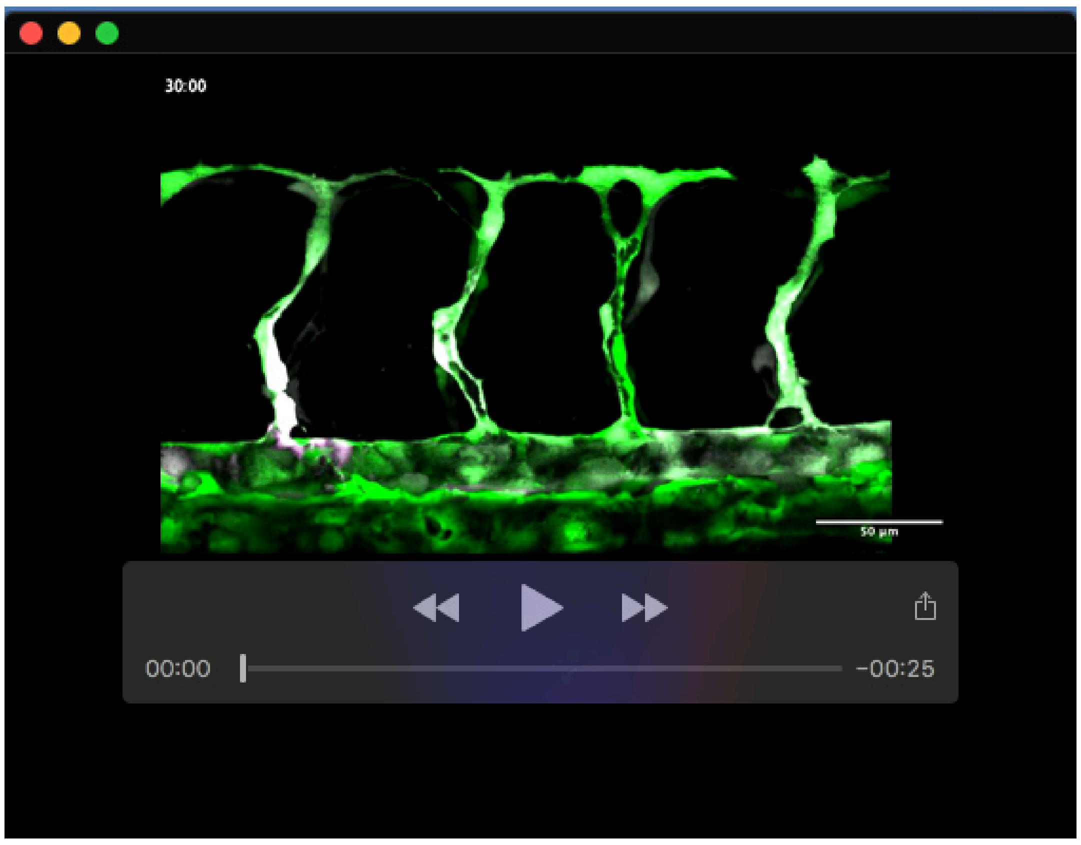

Movie 1. Time lapse movie of *MO-CTL* (5ng) *Tg(-0.8flt1:RFP)*^{hu3333}*; TgBAC(flt4:Citrine)* embryo treated with 1X (0.014%) tricaine from 30 to 48 hpf. 15-minute interval between frames.

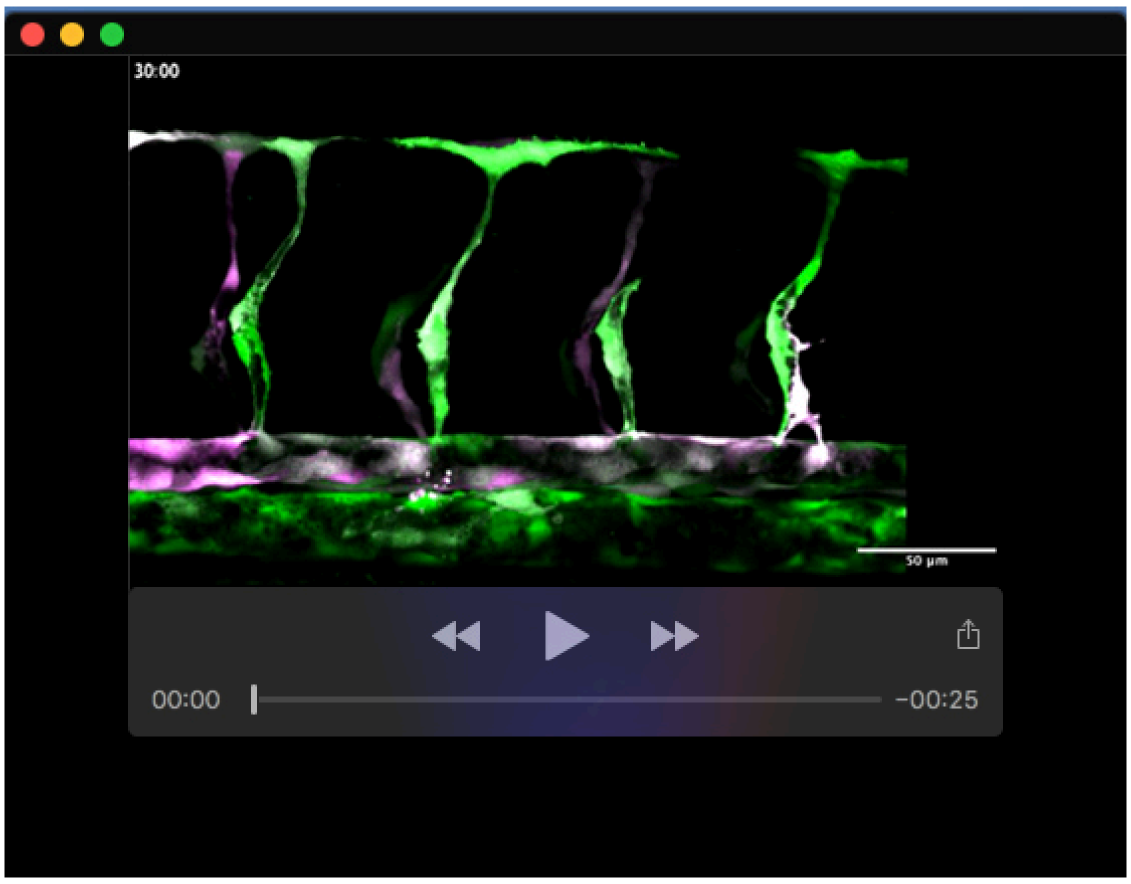

Movie 2. Time lapse movie of *MO-svep1* (5ng) *Tg(-0.8flt1:RFP)*^{hu3333}*; TgBAC(flt4:Citrine)* embryos treated with 1X (0.014%) tricaine from 30 to 48 hpf. 15-minute interval between frames.

Development • Supplementary information
