# Supplementary material for: Svep1 stabilises developmental vascular anastomosis in reduced flow conditions
Source: Development. 2022 Mar 25;149(6):dev199858. doi: 10.1242/dev.199858 (PMC8977097; doi:10.1242/dev.199858)
Supplement: Reviewer comments [file develop-149-199858_review_history.pdf]

Svep1 stabilises developmental vascular anastomosis in reduced flow conditions

Baptiste Coxam, Russell T. Collins, Melina Hussmann, Yvonne Huisman, Katja Meier, Simone Jung, Eireen Bartels-Klein, Anna Szymborska, Lise Finotto, Christian S. M. Helker, Didier Y. R. Stainier, Stefan Schulte-Merker and Holger Gerhardt
DOI: 10.1242/dev.199858

Editor: Steve Wilson

Review timeline

Original submission:	1 June 2021
Editorial decision:	9 June 2021
First revision received:	3 December 2021
Editorial decision:	27 January 2022
Second revision received:	3 February 2022
Accepted:	14 February 2022

Original submission

First decision letter

MS ID#: DEVELOP/2021/199858

MS TITLE: Svep1 stabilizes developmental vascular anastomosis in reduced flow conditions.

AUTHORS: Baptiste Coxam, Yvonne Padberg, Katja Maier, Simone Jung, Eireen Bartels-Klein, Anna Szymborska, Lise Finotto, Christian S.M Helker, Didier Y.R Stainier, Stefan Schulte-Merker, and Holger Gerhardt

I have now looked at this manuscript, the reviews and your response to them. You can access all files online: please go to BenchPressand click on the 'Manuscripts with Decisions' queue in the Author Area.

As the referees express considerable interest in your work, we will be happy to consider the manuscript for publication in Development after you have revised the study along the lines you outline in your response to the reviews from Review Commons. Please also note that Development will normally permit only one round of major revision.

Please attend to all of the reviewers' comments and ensure that you clearly highlight all changes made in the revised manuscript. Please avoid using 'Tracked changes' in Word files as these are lost in PDF conversion. I should be grateful if you would also provide a point-by-point response detailing how you have dealt with the points raised by the reviewers in the 'Response to Reviewers' box. If you do not agree with any of their criticisms or suggestions please explain clearly why this is so.

© 2022. Published by The Company of Biologists under the terms of the Creative Commons Attribution License (https://creativecommons.org/licenses/by/4.0/).

First revision

Author response to reviewers' comments

Point-by-point response:

Please find our detailed responses following each point highlighted by "Answer" and blue text highlight

Reviewer #1 (Evidence, reproducibility and clarity (Required)):

SUMMARY

This MS tackles a largely unknown topic of vessel formation: how vessels anastomose and lumenise.
The authors demonstrate that a matrix protein svep1 produced by neural tube during zebrafish embryogenesis plays a key role with blood flow to orchestrate anastomose formation. Actually in absence of this protein concomitantly with blood flow reduction results in significant decrease of lumenised DLAV segments.

In absence of svep1 they observed an expansion of apelin positive endothelial cells connected with a defect in tip/stalk cell specification. Interestingly the phenotype is amplified by blocking the kinase activity of VEGFR2

MAJOR COMMENTS

The most solid evidence on the role of blood flow in cooperating with svep1 relies on the use of tricaine, which reduces heart contractility. Interestingly the authors report some data by using embryo lacking cardiac troponin T2. In my opinion I suggest the author to better analyze the phenotype obtained by the deletion of svep1 together a dose-dependent reduction of tnnt2. This approach is more elegant and physiologic than the use of a chemical compound. Furthermore this approach will allow to better analyze the relations ship between blood flow and the expression of svep1 in neural tube. It should be relevant to establish a sort of flow threshold required to dampen lumenisation.

Answer: We appreciate the comment and have previously attempted to titrate the *tnnt2* morpholino as published to achieve a graded reduction in blood flow. In our hands, this has not proven to be a robust approach, and titration does not reproducibly lead to gradual reduction of flow. Importantly, our data does not support there being a threshold response in this process. We did not find that all intersegmental vessels lumenise and then stop when flow drops below a critical rate. Rather, there are progressively fewer lumenised segments as flow is reduced. This is evident as even with 2x tricaine in *svep1* morphants some segments are still able to lumenise. This may also relate to previous reports of two distinct mechanisms of lumen formation during DLAV formation, one driven by blood pressure, and the second by independent vacuole formation and their coalescence (so-called type 2 lumenisation).

To further improve the findings here reported I suggest to analyze the expression of klf2, which is a well known mechano-sensor of blood flow in several animal species including zebrafish.

Answer: We have performed this analysis and included the new data in the revised Figure 2H. The data demonstrate profound reduction in *klf2* expression in ISVs in line with the DLAV defects, but no changes in *klf2* expression in the perfused main axis vessels. The results are included in the text and suggest that flow-sensing per se is not affected.

It's likely that apelin is relevant in the observed phenotype. Which is the phenotype of a double mutant lacking both apl and svep1? Is there a direct influence of blood flow on apl expression?

© 2022. Published by The Company of Biologists under the terms of the Creative Commons Attribution License (https://creativecommons.org/licenses/by/4.0/).

Regarding *apelin* expression, we showed in the orginal figure 4H, I, that its expression is unchanged under 1x and 2x tricaine treatment. Therefore it seems that blood flow changes do not directly affect *apelin* expression.
[Regarding the interaction between apelin and svep1, the authors provided data to the referee in confidence; these are part of a current ongoing investigation and have therefore been removed from this file.]

Is there any suggestion that this mechanism is operative in mammalian?

Answer: This is an interesting question and certainly relevant for follow-up studies. At present, we can only speculate on a possible connection with flow, given that human SVEP1 mutations have recently been associated with artherosclerosis and increased blood pressure. This is also true in preeclampsia, but direct evidence for a role of SVEP1 in stabilizing vessel connections is currently missing. We mention these interesting findings in the last paragraph of the revised discussion. However, whether the anastomosis defect we identify is conserved remains to be seen.

Reviewer #1 (Significance (Required)):

The data here reported might represent a step forward in the field because a new mechanism is suggested.

The interest is sufficiently broad.

Reviewer #2 (Evidence, reproducibility and clarity (Required)):

Summary:

The authors demonstrated that loss of svep1 in zebrafish contributed to defective anastomosis of intersegmental vessels, in addition, such Svep1 acted synergistically with blood flow to modulate vascular network formation in the zebrafish trunk.

Major comments:

The expression of svep1 is localized in neurons of neural tube, dorsal epithelial cells (as indicated by transgenic zebrafish) and ventral somite boundary (as indicated by in situ) but is excluded from endothelial cells nor the vasculature. It remains puzzling and the authors have not addressed this very reason of how a gene that is expressed in non-vascular tissue play a crucial role in vessel anastomosis, ie DLAV, ISV lumenization, during angiogenesis. As the entire story of this svep1 is related to its function in angiogenic sprout and lumen formation of vascular tissues, it will be helpful for reader to be able to put the pieces together of how such gene may be functionally involved in such angiogenic process.
Previous publication of this gene involved in lymphoangiogenesis, as in this manuscript the authors could provide more evidence of how such gene and its localized expression contribute to different tissue in the vascular system, ie DLAV, instead of the neural tube, dorsal epidermis or ventral somite boundary.

Answer: We appreciate the wish to understand exactly how non-endothelial expression of Svep1 causes an endothelial phenotype selectively under reduced flow conditions. The very nature of this new phenotype requires analysis in vivo, and cannot easily be transferred to an ex vivo assay. Therefore, selective loss of function in different cell populations is not easily available. More importantly, the interpretation of such efforts, when mosaic, are marred with issues. At this point, we feel that full molecular characterization of how Svep1 affects endothelial cells during anastomosis will require entirely new approaches and lies beyond what can be achieved in this manuscript. Current efforts to identify binding partners and to zero in on which of the many domains of Svep1 may be involved are underway. However, given the discrete nature of the phenotype in conjunction with flow, this is, as stated above, beyond the scope of the current manuscript. We have attempted to make the discussion clearer regarding this aspect of the work, in line with the other comments by the reviewers.

Another puzzling point is that tricaine is the center of the subject in this study. As the authors

© 2022. Published by The Company of Biologists under the terms of the Creative Commons Attribution License (https://creativecommons.org/licenses/by/4.0/).

claim that tricaine-dependent blood flow reduction synergistically augmented the effect of svep1 deficiency. However, tricaine is known acting on neural voltage-gated sodium channels, whether svep1 function was affected by tricaine in the neural tissues and possibly its expression, the authors could provide more explanation and argument in the discussion.

Answer: We agree, this is an interesting and relevant point in particular as Svep1 is expressed by neural structures. In order to test a possible contribution of blocking neural activity, we have investigated whether selectively blocking neural function without disturbing heart activity can recapitulate the phenotype caused by tricaine in *svep1* KD conditions. We selectively blocked neuronal activity but not cardiac function by alpha-bungarotoxin mRNA injection (Swinburne, I.A., et al., Improved Long-Term Imaging of Embryos with Genetically Encoded alpha-Bungarotoxin. PLoS One, 2015. 10(8): p. e0134005.), and quantified DLAV gap formation and lumenisation in comparison to, and in combination with, tricaine treatment [new revised Figure 3C and D]. alpha-bungarotoxin had no effect on DLAV formation or lumenisation on its own, and did not augment the tricaine and *svep1* loss-of-function effects. These new results suggest that the observed sensitization to *svep1* loss-of-function by tricaine is attributable to its effects on blood flow.

It is unclear on p12 "These results suggest that while svep1 loss-of-function produces a cardiac defect that enhances the effect of tricaine on reducing blood flow, svep1 has an additive effect in modulating blood vessels anastomosis" that svep1 deficiency enhances the effect of tricaine leading to reduced blood flow, however, it is not accurate to state that svep1 loss-of-function produces a cardiac defect. It is not sure if the effect of svep1 was actually neural rather than cardiovascular tissue, for example, tricaine acts on neural voltage-gated sodium channel that slowing down heart beat. Whether the authors can explore the possibility that svep1 function in neural rather than cardiovascular tissues, may be discuss why the authors think svep1 enhances the blood flow defect (tnnt2a knockdown or tricaine) on angiogenesis such as DLAV phenotype.

Answer: As mentioned above, we used a-bungarotoxin mRNA injection to clarify this question.

On p13, the authors stated that svep1 expression was inhibited by reduced blood flow, however, is it really the effect of reduced blood flow or caused by the chemical tricaine? If tnnt2a knockdown showed a similar phenotype, then it may be more convincing.

Answer: The fact that *tnnt2* KD shows a similar phenotype to tricaine treatment in causing DLAV defects is shown in the original supplementary figure 2. This already suggests that flow reduction rather than a chemical effect of tricaine on neurons is causative. In the revised version we further support this finding by demonstrating that in contrast, selective blockage of neural function with a-bungarotoxin does not show the effects seen with tricaine.

Minor comments:

The work on "svep1 loss-of-function and knockdown are rescued by flt1 knockdown" was beautifully done and it is very clear and convincing.

The last two sections, "Vegfa/Vegfr signalling is necessary for ISV lumenisation maintenance and DLAV formation" and "Vegfa/Vegfr signalling inhibition exacerbates svep1 loss-of-function DLAV phenotype in reduced flow conditions" are more related to the flt1 knockdown phenotype. These 3 different sections are actually related in the sense that the rescue phenotype should be explained in the vegf signaling pathway. They are better off to discuss more cohesively about this vegf pathway that will help readers to appreciate more their work in svep1.

Answer: We agree and have unified the discussion to make this clearer.

Reviewer #2 (Significance (Required)):

This manuscript of svep1 in zebrafish provides new insight in angiogenesis, particularly in development of vessel anastomosis in zebrafish embryo, is very significant in the field and readers who are interested in angiogenesis and zebrafish development, including myself.

© 2022. Published by The Company of Biologists under the terms of the Creative Commons Attribution License (https://creativecommons.org/licenses/by/4.0/).

Reviewer #3 (Evidence, reproducibility and clarity (Required)):

This manuscript reports that the secreted extra-cellular matrix protein Svep1 plays a role in vascular anastomosis during developmental angiogenesis in zebrafish. Further, the study demonstrates that flow and Svep1 modulate the vascular network in a synergistic fashion. This is a high quality manuscript presenting novel data which compellingly support the conclusions that are made. I have no suggestions for further experimentation but list minor points below.

1. The final paragraph of Discussion is underdeveloped in that it claims regulation of phenotypic robustness in angiogenesis and its failure promises crucial insights into the mechanisms causing breakdown of vascular homeostasis in human disease. However, this issue is not pursued in any substantial way in Discussion. For example, are there known mutations in humans which lead to anastomosis defects and, if so, do any of them relate to the molecules or signaling pathways which are the subject of this manuscript?

Answer: We agree with the wish to see more substantial discussion of the issue of phenotypic robustness and potential links to human disease. We have expanded and developed this discussion including the interesting findings in humans that SVEP1 missense variants are associated with coronary artery disease and elevated blood pressure, missense mutations are related to outcome in septic shock through effects on endothelial inflammatory markers, and profound downregulation of SVEP1 mRNA was observed in cytotrophoblasts in preeclampsia. These findings although not direct evidence for a conserved role of our finding in zebrafish, raise the possibility that SVEP1 loss-of - unction is associated with vessel rarefaction in endothelial dysfunction in human disease. Whether the actual mechanism is indeed conserved will be tested in future work.

2. There are typographical errors in the text so a further proof-read is required.
Answer: thank you, we have carefully proof read and edited the manuscript, including the abstract to improve readability and make sure we correct all errors.

Reviewer #3 (Significance (Required)):

This manuscript provides an incremental conceptual advance in our understanding of the molecular mechanisms responsible for vascular anastomosis during developmental angiogenesis. The manuscript will be of interest to developmental biologists and vascular biologists.

My field of expertise pertains to angiogenesis and lymphangiogenesis in the setting of cancer and other diseases. I am not a developmental biologist.

Second decision letter

MS ID#: DEVELOP/2021/199858

MS TITLE: Svep1 stabilizes developmental vascular anastomosis in reduced flow conditions.

AUTHORS: Baptiste Coxam, Russell Thomas Collins, Melina Hussmann, Yvonne Huisman, Katja Meier, Simone Jung, Eireen Bartels-Klein, Anna Szymborska, Lise Finotto, Christian S.M Helker, Didier Y.R Stainier, Stefan Schulte-Merker, and Holger Gerhardt

Apologies for the delay in considering your manuscript due to waiting on one of the original referees. As this referee still hasn't managed to submit their review, I have made a decision based on the two the referees reports I have received. The referees' comments are appended below, or you can access them online: please go to BenchPressand click on the 'Manuscripts with Decisions' queue in the Author Area.

The referees are happy with your revisions and there is just one minor issue for you to consider before we proceed to publication.

© 2022. Published by The Company of Biologists under the terms of the Creative Commons Attribution License (https://creativecommons.org/licenses/by/4.0/).

Reviewer 1

Advance summary and potential significance to field

This manuscript of svep1 in zebrafish provides new insight in angiogenesis particularly in development of vessel anastomosis in zebrafish embryo, is very significant in the field.

Comments for the author

This revised manuscript is substantially improved and have addressed critical issues raised by the reviewers.

Reviewer 2

Advance summary and potential significance to field

According to my previous revision I guess these findings innovative in vascular field

Comments for the author

The authors carefully performed the revision. Unfortunately many questions are still open and I agree with the authors that some points are difficult to explain. I suggest to include the in the discussion a paragraph to clearly underline the limit of their findings. I think this suggestion could be useful for other points (referee 2), which have been partially clarified

---

Second revision

Author response to reviewers' comments

Reviewer 1

We would like to thank the reviewer for the positive comments.

Reviewer 2

We appreciate the wish to see the limitations of the work mentioned in the discussion. The revised manuscript now includes a short statement to this regard in the penultimate paragraph.

Third decision letter

MS ID#: DEVELOP/2021/199858

MS TITLE: Svep1 stabilizes developmental vascular anastomosis in reduced flow conditions.

AUTHORS: Baptiste Coxam, Russell Thomas Collins, Melina Hussmann, Yvonne Huisman, Katja Meier, Simone Jung, Eireen Bartels-Klein, Anna Szymborska, Lise Finotto, Christian S.M Helker, Didier Y.R Stainier, Stefan Schulte-Merker, and Holger Gerhardt
ARTICLE TYPE: Research Article

Apologies for the delay in responding. I am happy to tell you that your manuscript has been accepted for publication in Development, pending our standard ethics checks.

© 2022. Published by The Company of Biologists under the terms of the Creative Commons Attribution License (https://creativecommons.org/licenses/by/4.0/).
